# Characterization of the Electrophysiologic Remodeling of Patients With Ischemic Cardiomyopathy by Clinical Measurements and Computer Simulations Coupled With Machine Learning

**DOI:** 10.3389/fphys.2021.684149

**Published:** 2021-07-14

**Authors:** Konstantinos N. Aronis, Adityo Prakosa, Teya Bergamaschi, Ronald D. Berger, Patrick M. Boyle, Jonathan Chrispin, Suyeon Ju, Joseph E. Marine, Sunil Sinha, Harikrishna Tandri, Hiroshi Ashikaga, Natalia A. Trayanova

**Affiliations:** ^1^Section of Electrophysiology, Division of Cardiology, Johns Hopkins Hospital, Baltimore, MD, United States; ^2^Department of Biomedical Engineering, The Institute for Computational Medicine, Johns Hopkins University, Baltimore, MD, United States

**Keywords:** ischemic cardiomyopathy, action potential duration restitution, genetic algorithms, unsupervised machine learning, patient-derived disease-specific action potential models

## Abstract

**Rationale:**

Patients with ischemic cardiomyopathy (ICMP) are at high risk for malignant arrhythmias, largely due to electrophysiological remodeling of the non-infarcted myocardium. The electrophysiological properties of the non-infarcted myocardium of patients with ICMP remain largely unknown.

**Objectives:**

To assess the pro-arrhythmic behavior of non-infarcted myocardium in ICMP patients and couple computational simulations with machine learning to establish a methodology for the development of disease-specific action potential models based on clinically measured action potential duration restitution (APDR) data.

**Methods and Results:**

We enrolled 22 patients undergoing left-sided ablation (10 ICMP) and compared APDRs between ICMP and structurally normal left ventricles (SNLVs). APDRs were clinically assessed with a decremental pacing protocol. Using genetic algorithms (GAs), we constructed populations of action potential models that incorporate the cohort-specific APDRs. The variability in the populations of ICMP and SNLV models was captured by clustering models based on their similarity using unsupervised machine learning. The pro-arrhythmic potential of ICMP and SNLV models was assessed in cell- and tissue-level simulations. Clinical measurements established that ICMP patients have a steeper APDR slope compared to SNLV (by 38%, *p* < 0.01). In cell-level simulations, APD alternans were induced in ICMP models at a longer cycle length compared to SNLV models (385–400 vs 355 ms). In tissue-level simulations, ICMP models were more susceptible for sustained functional re-entry compared to SNLV models.

**Conclusion:**

Myocardial remodeling in ICMP patients is manifested as a steeper APDR compared to SNLV, which underlies the greater arrhythmogenic propensity in these patients, as demonstrated by cell- and tissue-level simulations using action potential models developed by GAs from clinical measurements. The methodology presented here captures the uncertainty inherent to GAs model development and provides a blueprint for use in future studies aimed at evaluating electrophysiological remodeling resulting from other cardiac diseases.

## Introduction

Ischemic cardiomyopathy (ICMP) patients are at high risk for malignant arrhythmias (Moss et al., 1996; Moss et al., 2002; Bardy et al., 2005), largely due to tissue heterogeneity from infarct-related fibrosis (structural substrate) (Haqqani et al., 2009; Roes et al., 2009; Nakahara et al., 2010; Estner et al., 2011), and electrophysiologic (EP) remodeling of the myocardium (Pu and Boyden, 1997; Yue et al., 1998; Jiang et al., 2000; Huang et al., 2001; Camelliti et al., 2004; Dun et al., 2004; Isidoro Tavares et al., 2007; Liu et al., 2007). The role of the structural substrate of ICMP in arrhythmogenesis has been well characterized in clinical studies (Haqqani et al., 2009; Roes et al., 2009; Nakahara et al., 2010). However, the EP remodeling and its contribution to the dynamic EP behavior of the myocardium has not been established in ICMP patients yet.

The first goal of this study is to characterize the EP remodeling of the arrhythmogenic substrate in ICMP patients, and specifically changes in electrical restitution. Action potential duration restitution (APDR) is an important EP property that contributes to tissue-level EP dynamics, and is critical in arrhythmogenesis (Banville and Gray, 2002; Watanabe et al., 2002; Wu et al., 2002; Pak et al., 2004; Yuuki et al., 2004; Selvaraj et al., 2007; Benoist et al., 2012). The steepness and dispersion of APDR are mechanistically linked to development of malignant arrhythmias in pre-clinical studies (Banville and Gray, 2002; Watanabe et al., 2002; Wu et al., 2002; Yuuki et al., 2004; Benoist et al., 2012) and are associated with increased arrhythmic risk in limited clinical studies (Pak et al., 2004; Selvaraj et al., 2007). Steep APDR is associated with the development of APD alternans. APD alternans refer to alternating APD between two subsequent beats with one beat having a longer APD and the other having a shorter APD and are mechanistically linked to development of arrhythmias (Tse et al., 2016). The APDR of the surviving left ventricular (LV) myocardium of ICMP patients, and its contribution to the tissue-level EP dynamics is, however, yet to be described. Previous studies have evaluated APDR in patients with structural heart disease (SHD) (Koller et al., 2005; Selvaraj et al., 2007; Dorenkamp et al., 2013). However, these studies assessed APDR at the right ventricular (RV) apex, septum, or outflow track. APDR of the RV is significantly different from that of the LV (Srinivasan et al., 2016). Furthermore, although these studies included ICMP patients they report combined results for patients with any SHD. Knowledge of the APDR of the surviving LV myocardium in ICMP patients and its contribution to tissue-level EP dynamics is important for understanding the pro-arrhythmic substrate of ICMP patients.

The second goal of this study is to develop a methodology that allows for action potential model development from experimental APDR data, that can subsequently be used in Virtual Heart modeling, to improve clinical risk stratification and ablation planning. Virtual Heart modeling is a powerful platform for non-invasive ventricular tachycardia (VT) risk assessment (Arevalo et al., 2016; Deng et al., 2016), localization (Ashikaga et al., 2013), and ablation planning (Prakosa et al., 2018) in ICMP patients. Virtual Heart modeling uses 3D reconstructions of the heart derived from cross-sectional imaging to perform simulations and assess for the emergence of arrhythmias *in silico*. Incorporating the APDR of non-infarcted myocardium in Virtual Heart models of ICMP patients is of critical importance: First, current cardiac action potential models have been derived from pre-clinical data (Niederer et al., 2009), and do not capture the APDR of the non-infarcted myocardium of ICMP patients. Second, in sensitivity analysis, APD is important in determining the location of VT in ICMP patients (Deng et al., 2019), as well as the trajectory of re-entrant drivers in fibrillatory rhythms (Deng et al., 2017). Third, incorporating APDR in Virtual Heart models will: (1) provide mechanistic insight on how the remodeled myocardium contributes to the initiation and maintenance of VT, and development of ventricular fibrillation (VF); and (2) allow for development of new risk stratification and therapeutic strategies based on more accurate computer-based simulations.

Specifically, our aims are: (1) to clinically characterize the APDR of the surviving LV myocardium of ICMP patients and compare it to the APDR of structurally normal left ventricles (SNLV); (2) to develop the methodology to construct action potential models based on clinically obtained cohort-specific APDRs using a custom-developed genetic algorithm (GA); (3) to compare the emerging EP dynamic behaviors between ICMP and SNLV in cell- and tissue-level computer simulations using the GA-derived action potential models; and (4) to capture the uncertainty in the GA-derived action potential models and in the resulting cell-level and tissue-level EP behavior by combining computer simulations with unsupervised machine learning. The results of this study have important implications because they improve our understanding on the EP substrate of ICMP patients and provide a methodology for calibration of cardiac action potential models account for the uncertainty related to model development from clinical data. Such models can be used for the development of personalized risk-stratification and guidance of ablative strategies, contributing to the ongoing efforts towards precision medicine in cardiology.

## Materials and Methods

### Overview of the Study Approach

To assess the activation-repolarization dynamics in ICMP compared to that of SNLV, we performed programmed electrical stimulation in patients undergoing left-sided catheter ablation procedures. We used the corresponding unipolar electrograms (UniEGMs) to obtain APDR curves with the help of a customized signal processing approach. We then utilized the clinically obtained APDRs to construct populations of action potential models of ICMP and SNLV patients, employing a custom-developed GA. We used unsupervised machine learning to capture the uncertainty in model development from clinical data. We then used the developed action potential models to characterize the dynamic pro-arrhythmic substrate of ICMP patients.

### Patient Enrollment

We prospectively enrolled consecutive patients undergoing left-sided catheter ablation procedures at the Johns Hopkins Hospital. Criteria for inclusion were patients older than 18 years old undergoing any clinically indicated left-sided catheter ablation procedure that had either ICMP or SNLV. We required that all participants had echocardiographic assessment within 1 year of the procedure day. The presence of ICMP was ascertained by a positive history for coronary artery disease and decreased left ventricular systolic function on echocardiogram, with wall motion abnormalities consistent with the distribution of coronary artery disease. Exclusion criteria were: patients younger than 18 years old, pregnancy, presentation with electrical storm, presence of cardiomyopathy other than ischemic, need for inotropic support prior to the procedure, need for mechanical circulatory support before or during the procedure and patients with congenital heart diseases. All patients gave informed consent to participate in the study. Enrollment target was a total of 20 patients (10 ICMP and 10 SNLV patients).

### Clinical Protocol for Assessment of Cardiac Activation-Repolarization Dynamics

We evaluated activation-repolarization dynamics in the non-infarcted myocardium of ICMP patients and the myocardium of patients with SNLV by pacing at a fixed cycle length at progressively decreasing cycle lengths, 20 min after completion of the clinical ablation procedure. Specifically, we placed either a multi-electrode mapping catheter (Pentaray 4-4-4; Biosense Webster or LiveWire; Abbott), or a standard 4-mm tip ablation catheter with 2 mm inter-electrode spacing (Thermocool; Biosense Webster, Inc.) in an area (remote from scar in ICMP patients) with a bipolar signal voltage amplitude >1.5 mV. For each patient only one of either an ablation or a multi-electrode catheter was used for data collection. A multi-electrode catheter was used if the operating electrophysiologist had deemed it necessary for clinical/procedural purposes. We then paced at a fixed cycle length the patient from a catheter placed in the RV apex for 20 beats at cycle lengths decrementing from 600 to 350 ms with a step of 50 ms. These pacing maneuvers were performed with intervals of 15 s to a few minutes, allowing for blood pressure recovery in case the blood pressure dropped with fast pacing. We recorded UniEGM from the mapping or ablation catheter during burst pacing (CardioLab Recording Systems; GE Healthcare). Signals were recorded from all electrodes at a fixed catheter location in the left ventricle and catheter stability during recordings was confirmed fluoroscopically. The sampling frequency of the recorded signal was 977 Hz. The recorded signal was filtered using a high-pass filter at 0.05 Hz, a low-pass filter at 150 Hz and a notch filter at 60 Hz (CardioLab Recording Systems; GE Healthcare).

### Signal Processing to Obtain APDR Curves

To construct clinically obtained APDR curves, we approximated the action potential duration (APD) at each pacing cycle length and location using the activation-recovery interval (ARI) method on the recorded UniEGMs. We analyzed the electrograms from each recording site of the multi-electrode catheter separately. The ARIs at each cycle length was assessed using the Wyatt method (Millar et al., 1985; Haws and Lux, 1990; Yue et al., 2004; Coronel et al., 2006; Potse et al., 2009). ARI was defined as the interval between the steepest negative slope (*min dV/dt*) of the UniEGM ventricular activation component and the steepest positive slope (*max dV/dt*) of the UniEGM ventricular repolarization component. ARIs obtained from experimental and clinical measurements have been extensively validated as a surrogate measure of APD (Millar et al., 1985; Haws and Lux, 1990; Yue et al., 2004; Coronel et al., 2006; Potse et al., 2009).

To maximize accuracy and reproducibility of ARI measurement, we extracted ARI from the recorded UniEGMs using a custom-made, semi-automatic approach that combined: (a) signal post-processing to attenuate experimental noise and improve ARI detection accuracy (van Duijvenboden et al., 2015); and (b) a template-based method to enhance reproducibility in the presence of temporal lability of ARI intervals (Berger et al., 1997; Berger, 2003). The details and a schematic of this approach can be found in Supplementary Materials and Methods and Supplementary Figure 1.

To assess the APDR intercept and slope of the myocardium of patients with ICMP and SNLV, for each recording, we plotted the ARI of each beat against its preceding diastolic interval (DI), constructing a clinically obtained APDR curve. We excluded from analysis the first three beats and the last beat of each pacing burst, to remove UniEGM waveforms that were not at steady-state. A logarithmic curve was fitted to the measured ARI and its preceding DI. Since ARI approximates APD (Millar et al., 1985; Haws and Lux, 1990; Yue et al., 2004; Coronel et al., 2006; Potse et al., 2009), the intercept and slope of the fitted curve approximate the intercept and slope of the average APDR at the location of the recording. To control for heteroskedasticity that is inherent in these data, we fitted these curves using a bisquare robust regression approach (MATLAB, Natick, MA, United States). Only recordings that yielded a regression coefficient of determination *R*^2^ > 50% were used in analysis.

### Statistical Analysis

Baseline characteristics are presented stratified by ICMP/SNLV status. Continuous variables are presented as median (inter-quartile range) and categorical variables as count (percentage). Comparisons between baseline characteristics in the ICMP and SNLV groups are performed using the Wilcoxon rank-sum test for continuous variables and the Pearson’s Chi-squared or Fisher’s exact test for categorical variables. ARI and DI for each pacing cycle length is reported as mean ± SEM. To compare APDR intercept and slope between ICMP and SNLV groups we used linear mixed-effects models. We used this approach to account for the fact that for each patient we had multiple measurements (multiple simultaneous recordings per patient). ICMP/SNLV status was introduced as a fixed effect in the model and patient number as a random effect. With this approach each electrode recording was nested within each patient and each patient was classified by the ICMP/SLNLV status. Robust variance-covariance matrix estimators were used. We performed sensitivity analysis by repeating the mixed model analysis after excluding patients with atrial fibrillation, patients taking any membrane active anti-arrhythmic medication (class I, class III, or ranolazine), the patient with the highest and lowest APDR slope, women (since we were unable to enroll women in the ICMP cohort), and restitution curves that had a regression coefficient of determination <60, 70, 80, and 90%. The effect of patient diagnosis [premature ventricular complex (PVC) vs VT] and catheter selection (Pentarray vs Livewire, vs ablation catheter) was examined, by introducing these as fixed effect co-variates in the mixed model analysis. All *p*-values are two-sided, and the statistical significance criterion was set at an alpha of 0.05. We performed all statistical analysis using Stata version 14.2 (StataCorp, College Station, TX, United States).

### Development of Action Potential Models Incorporating Patient-Derived APDR Using GA

The APDRs obtained as described above are surrogates of cellular activation-repolarization dynamics. In order to develop ICMP and SNLV action potential models we used average APDR derived from the ICMP and SNLV cohorts. Next, we used these APDRs to construct populations of action potential models of ICMP and SNLV patients that capture APDR properties. Those action potential models were, in turn, used to characterize the contribution of APDR to the emerging tissue-level EP behavior and arrhythmogenesis in ICMP.

We used GA to construct the action potential models. GAs are biologically inspired metaheuristics that are appropriate for fitting action potential models to experimental measurements, since action potential models have parameter spaces that are highly nonlinear, frequently discontinuous, with multiple local minima. To proceed with action potential model development from the clinical data using GA, we needed a baseline action potential model with small number of variables and parameters that would allow for computationally tractable execution of the GA. For this, the endocardial formulation of the Bueno-Orovio-Fenton-Cherry (BOFC) action potential model was selected (Bueno-Orovio et al., 2008). The BOFC (also known as the “Minimal Ventricular Model”) is a phenomenological action potential model, that can accurately replicate the ventricular myocyte electrophysiologic behavior, while having a small number of variables and parameters (4 state variables and 28 parameters). Details regarding the numerical aspects of our GA algorithm can be found in Supplementary Materials and Methods.

We designed and optimized a GA that incorporates the clinically obtained APDR curves in the BOFC model using the local-iterative approach (Groenendaal et al., 2015; Krogh-Madsen et al., 2017). A complete discussion on the design, optimization, and implementation of the GA can be found in Supplementary Materials and Methods and Supplementary Figure 2A. Briefly, GAs iteratively assesses the “fitness” of a population of model parameters and uses the principles of natural selection to derive the optimal parameter set. Specifically, our GA generated a population of 1512 random parameter sets. These parameters were used in cell-level simulations, using a dynamic restitution protocol, to produce an APDR. The simulation-derived APDR was compared to the one obtained from the clinical measurements in SNLV or ICMP patients and the error between these two curves was expressed as mean absolute error. The GA sought a parameter set that minimizes this error.

### Classification of the Populations of GA-Derived Models Using Machine Learning

Optimization problems of non-linear differential equations, such as the ones that describe cardiac action potential, do not have unique solutions, given a finite amount of data. Consequently, GAs yield a population of suitable derived models. These derived models may have different emergent behaviors when used in tissue-level simulations. Executing simulations with all derived models would capture the variability with respect to emergent behaviors but would be computationally not tractable. To circumvent this, we used an unsupervised machine learning approach to explore the entire population of derived models and group models based on the degree of dissimilarity with respect to their parameter values.

We explored the hierarchical organization and evaluated for the presence of clustering in the population of derived models yielded by our GA using agglomerative hierarchical clustering (AHC) (Zepeda-Mendoza and Resendis-Antonio, 2013), an unsupervised machine learning approach. AHC groups data over a variety of scales by creating a hierarchical cluster tree (dendrogram). We then cut the hierarchical tree such that the data are partitioned in the most dissimilar clusters. This resulted in the initial parameter space to be divided in the most-dissimilar parameter spaces. The centroid of each parameter space was used in cell-level and tissue-level simulations. To understand the uncertainty related to model development from clinical data, we performed cell-level and tissue-level simulations, using the best-fit models derived from the GA and the centroids of the parameter spaces derived from AHC.

### Cell-Level Simulations to Evaluate the Dynamic Onset of APD Alternans

To characterize the pro-arrhythmic substrate of ICMP patients, we performed cell-level and tissue-level simulations using the best fit and each of the most dissimilar action potential models derived from our GA. In cell-level simulations a single cell was paced using a decremental dynamic restitution protocol to explore the onset of APD alternans. The cell was paced for 10 beats at cycle lengths starting at 620 ms and decrementing to 260 ms with a step of 5 ms. We used the last two beats of each 10-beat burst for APD assessment. We defined APD at the 10% of the peak action potential voltage (APD90). We constructed bifurcation plots of APD over cycle length and we marked the cycle length where APD alternans occurred. APD alternans were defined if the difference of the APD between two consecutive beat was greater or equal to 2 ms for two consecutive pacing cycle lengths. Earlier onset of APD alternans (i.e., with shorter cycle length) suggests a more proarrhythmic behavior.

### Tissue-Level Simulations to Characterize the Emerging EP Dynamic Behaviors in ICMP

To characterize the contribution of ICMP APDR to the dynamic EP behaviors emerging at the tissue level, we performed tissue-level simulations using the best fit and each of the most dissimilar action potential models derived by our GA. These simulations were performed on a 2 cm × 2 cm × 0.25 mm isotropic cardiac tissue slab. The cells were pre-paced of the slab for 100 beats at a cycle length of 600 ms to achieve steady-state. First, we performed a restitution protocol on tissue-level simulations using the same settings with what we describe in cell-level simulations. The purpose of this was to compare the APDR of the GA-derived models in tissue-level simulations with that of the cell-level simulations. We then performed a S1S2 cross-field stimulation protocol to assess for inducibility of sustained functional re-entry. Specifically, we delivered S1 as a single stimulus at the lower edge of the slab (area of stimulation: 2 cm × 0.5 mm × 0.25 mm) and S2 in a rectangular area in the lower left corner of the slab (area of stimulation: 1 cm × 1 cm × 0.25 mm). We tested S1S2 coupling intervals starting from 500 ms and decreasing to 50 ms by a step of 5 ms. The model was defined as inducible for a given S1S2 coupling interval, if sustained functional re-entry was induced and persisted for >2 s in simulation. As we conduction velocity was not assessed in our cohort, we executed the above-described tissue-level simulations over a wide range of conductivity values (from 0.001 to 0.012 in steps of 0.0005 Siemens/m). This conductivity range corresponds to the clinically observed conduction velocity range of 17–74 cm/s. For each conductivity value, we defined the range of S1–S2 coupling intervals that the cross-field simulation resulted in sustained functional re-entry. A wider range of coupling intervals that result in sustained functional re-entry suggests a more proarrhythmic behavior.

## Results

### Patient Characteristics

We enrolled 10 ICMP patients and 12 patients with SNLV in this study. Patient characteristics are summarized in Table 1. Mean age was 62–69 years old and males were over-represented in the ICMP cohort compared to the SNLV cohort (100 vs. 41%, respectively). Left ventricular ejection fraction was significantly lower (35 vs 60%, *p* < 0.001) and left ventricular end-diastolic diameter was significantly higher (5.6 vs 4.75 cm, *p* = 0.002) in ICMP patients compared to SNLV. All ICMP patients and 9/12 SNLV underwent ablation for VT or PVC. 3/12 patients with SNVL underwent AF ablation procedure. VT was the indication of the ablation procedure more frequently in ICMP patients compared to SNLV (80 vs 16.7%, *p* = 0.009). All ICMP patients and 50% with SNLV were on beta blockers. Four ICMP patients and one with SNLV were on amiodarone. A similar percentage of ICMP and SNLV patients had APDR data collected using a multi-electrode ablation catheter vs an ablation catheter (for multi-electrode catheter 60 vs 50%, *p* = 0.69, Supplementary Results).

**TABLE 1:** Baseline characteristics of patients with ICMP and SNLV.

	**SNLV (*N* = 12)**	**ICMP (*N* = 10)**	***p*-value**
Age (years)	62 (19)	69 (12)	0.098
Male (*n*, %)	5 (41)	10 (100)	0.005*
EF (%)	60 (7.5)	35 (25)	<0.001*
LVEDD (cm)	4.75 (0.55)	5.6 (0.9)	0.002*
VT (*n*, %)	2 (16.7)	8 (80)	0.009*
AAD class IB	0	1 (10%)	0.45
AAD class IC	2 (16.7%)	0	0.48
AAD class II	6 (50%)	10 (100%)	0.02*
AAD class III	1 (8.3%) 0	4 (40%)	0.14
AAD class IV	2 (16.7%)	0	0.48
CCB-DHP	2 (16.7%)	0	0.48
Ranolazine	0	2 (20%)	0.19
ACEI	2 (16.7%)	8 (80%)	0.008*
Digoxin	0	1 (10%)	0.45
Spironolactone	0	1 (10%)	0.45
Multi-electrode catheter	6 (50%)	6 (60%)	0.69

*AAD, anti-arrhythmic drugs; ACEI, angiotensin converting enzyme inhibitors; CCB-DHP, calcium channel blockers dihydropyridine class; EF, ejection fraction; ICMP, ischemic cardiomyopathy; LVEDD, left ventricular end-diastolic diameter; SNLV, structurally normal left ventricle; VT, ventricular tachycardia as an indication for the ablation procedure.*

The row labeled “Multi-Electrode Catheter” summarizes the number (%) of patients that had APDR data collected using a multi-electrode catheter (either Pentaray 4-4-4; Biosense Webster or LiveWire; Abbott). Values in bold represent p-values less than 0.05 which is the cut-off for statistical significance. *Denotes p-values less than 0.05 which is the cut-off for statistical significance.

### ICMP Patients Have a Steeper APDR Compared to Those With SNLV

**FIGURE 1:**
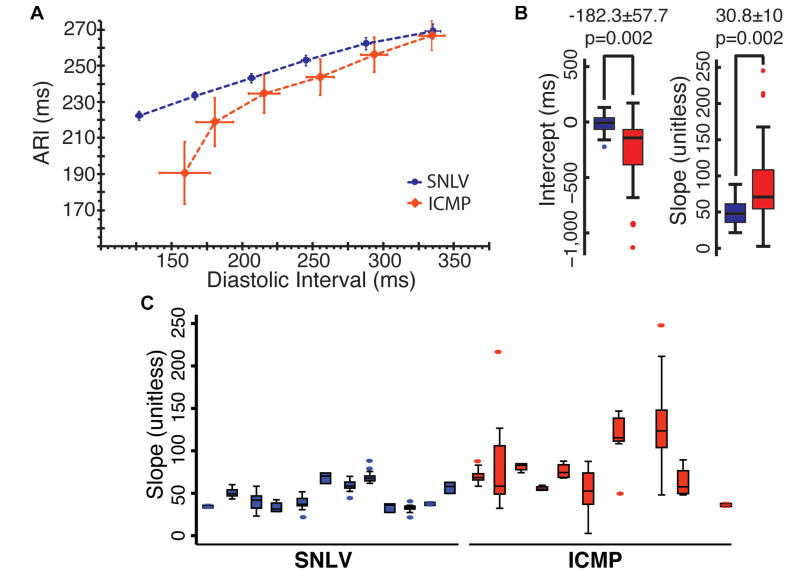
**(A)** ARI over DI in patients with SNLV (blue) and ICMP (red). Points represent mean values and error bars represent 95% confidence intervals. ARI, activation recovery interval in ms; DI, diastolic interval in ms. **(B)** Box-plots demonstrating the distribution of clinically obtained APDR intercept (in ms) and slope (unitless) in patients with SNLV (blue) and ICMP (red). Box-plots summarize the intercept and slope of the clinically obtained APDR curve fitted at each UniEGM recording of each patient. *p*-values are derived from mixed model analysis as described in the main text. **(C)** Box-plots of the clinically obtained APDR slope in each individual SNLV and ICMP patient, demonstrating the within- and between-subject variability of clinically obtained APDR slope.

A total of 231 APDR curves were used in this analysis (103 from ICMP). Mean ARI over mean DI in ICMP patients and SNVL is presented in Figure 1 and Table 2. When pacing at a cycle length of 600 ms, ARI was similar between SNLV and ICMP (269.1 ± 21.8 vs 266.8 ± 19.1 ms, respectively). The restitution curves separated when pacing at 500 ms with ARIs of 253.0 ± 15.6 and 243.8 ± 23.3 for SNLV and ICMP, respectively. The overall variability of ARI is significantly higher in ICMP patients compared to SNLV for the entire range of pacing cycle lengths (SD 19.1–40.4 vs 12.0–21.8 ms, Table 2).

**TABLE 2:** Activation recovery interval and diastolic interval during different pacing cycle lengths in patients with ICMP and SNLV.

**Pacing cycle length (ms**)	**SNLV**		**ICMP**	
	**DI (ms)**	**ARI (ms)**	**DI (ms)**	**ARI (ms)**
600	335.2 ± 29.0	269.1 ± 21.8	334.3 ± 21.1	266.8 ± 19.1
550	287.9 ± 18.1	262.2 ± 17.8	293.5 ± 21.9	256.2 ± 22.6
500	245.2 ± 16.9	253.0 ± 15.6	255.3 ± 22.7	243.8 ± 23.3
450	206.7 ± 13.2	243.2 ± 13.7	215.6 ± 25.5	234.6 ± 25.2
400	166.6 ± 11.6	233.4 ± 12.0	180.6 ± 30.7	218.8 ± 31.1
350	127.1 ± 13.2	222.3 ± 13.1	159.3 ± 41.9	190.6 ± 40.4

*Results are mean ± SD.*

*ARI, activation-recovery interval; DI, diastolic interval; ICMP, ischemic cardiomyopathy; SNLV, structurally normal left ventricle.*

The APDR curve fitted to the clinical data was significantly steeper in ICMP patients compared to SNLV. In mixed model analysis, ICMP patients had a significantly higher APDR slope [steeper by 30.8 ± 10 (38%), *p* = 0.002] and a significantly lower intercept (by 182.3 ± 57.7 ms, *p* = 0.002). Variance decomposition analysis demonstrated that APDR slope of ICMP patients had higher within-subject and between-subject variance compared to SNLV (within-subject SD of 29.1 vs 6.4 and between-subject SD of 29.1 vs 13.9 for ICMP and SNLV, respectively). Sensitivity analysis revealed that ICMP patients have a significantly steeper APDR slope compared to those with SNLV even after exclusion of patients with AF, patients taking any membrane-active antiarrhythmic medication, patients with the highest and lowest APDR slope, and women (difference in slope of APDR – SNLV 17.9–37.7, Supplementary Table 1). APDR remained significantly steeper in ICMP patients compared to SNLV even after adjusting the mixed-model analysis for catheter type (APDR slope difference: 27.3 ± 11.1, *p* = 0.014), or VT/PVC status (APDR slope difference: 26.0 ± 11.1, *p* = 0.02). Patients that underwent VT ablation had on average similar APDR intercept and slope with patients that underwent PVC ablation.

### ICMP Models Have Significantly Different Parameters Compared to SNLV Models

**FIGURE 2:**
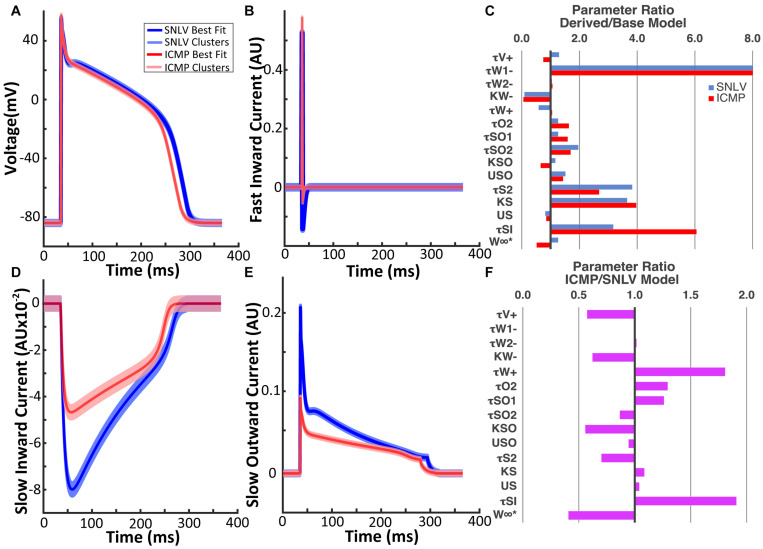
**(A)** Action potential waveform of the SNLV and ICMP models. **(B)** Fast inward current waveform of the SNLV and ICMP models. **(D)** Slow inward current waveform of the SNLV and ICMP models. **(E)** Slow outward current waveform of the SNLV and ICMP models. Waveforms in **(A,B,D,E)** are derived from cell-level simulations during pacing at 500 ms. Bold blue and red lines represent the waveforms of the best fit models, while light blue and red lines represent the wavefronts using the centroids of the six highest clusters. Jitter has been applied to the waveforms using the centroids of the six highest clusters to facilitate visualization. **(C)** Fold-difference of the parameters of SNLV (blue) and ICMP (red) compared to the endocardial parameter set of the baseline BOFC model. The parameter τ_*w*1^−^_ of SNLV and ICMP models is 16.6- and 16.7-fold higher than the baseline BOFC model but the bar chart has been truncated at 8 for better visualization of the remaining parameters. **(F)** Fold-difference of the parameters of ICMP compared to SNLV models.

The GA converged at 174 generations during SNLV model derivation and at 227 generations during ICMP model derivation (Supplementary Figure 2B). The action potential waveforms of the derived models are shown in Figure 2. Action potential biomarkers of the SNLV and ICMP model are presented in Table 3. Parameters describing the depolarization phase of the action potential are similar between the SNLV and ICMP model. APD is shorter for the ICMP compared to the SNLV model. The ionic currents of the SNLV and ICMP models are shown in Figure 2. The amplitude and duration of the fast inward current was similar between SNLV and ICMP models. The amplitude of the slow inward and slow outward currents was decreased in ICMP compared to SNLV.

**TABLE 3:** Action potential biomarkers for the base BOFC and derived SNLV and ICMP models.

**Model**	**RMP (mV)**	**Vmax (mV)**	**Tmax (ms)**	**dV/dt (V/s)**	**APD30 (ms)**	**APD50 (ms)**	**APD90 (ms)**	**Tri(90-30) (ms)**	**Tri(30/90)**	**Tri(90-50) (ms)**	**Tri(50/90)**
Base	−84.0	53.8	0.48	287.2	123.7	226.5	276.3	152.6	0.45	49.8	0.82
SNLV	−84.0	55.5	0.48	290.7	98.2	210.5	256.8	158.6	0.38	46.3	0.82
ICMP	−84.0	57.6	0.48	295.1	77.2	197.5	242.5	165.3	0.32	45.0	0.81

*Biomarkers were obtained by pacing the action potential models at 500 ms.*

*APD30, APD50, and APD90, action potential duration at 30, 50, and 90% repolarization; dV/dt, upstroke velocity; RMP, resting membrane potential; Tmax, time to maximal voltage upstroke; Tri(90-30), triangulation index defined as APD90-APD30; Tri(30/90), triangulation index defined as APD30/APD90; Tri(90-50), triangulation index defined as APD90-APD50; Tri(50/90), triangulation index defined as APD50/APD90; Vmax, maximal voltage of action potential upstroke. Tri(30/90) and Tri(50/90) are unitless.*

The parameters of the best fit model for SNLV and ICMP GA-derived models are presented in Supplementary Tables 2, 3, respectively. Overall, the parameters of GA-derived SNLV and ICMP were significantly different (up to 16.7-fold) compared to the baseline BOFC model. The parameters that deviated the most from the baseline BOFC model were τ_*w*1^−^_ (16.6 and 16.7 fold-change),τ_*si*_ (3.2 and 6.1 fold-change), *k_s* (3.7 and 4.0 fold-change), and τ_*s*2_ (3.8 and 2.7 fold-change for SNLV and ICMP, respectively, Figure 2C). τ_*w*1^−^_ controls the inactivation gate of the slow inwards depolarizing current,τ_*si*_, directly controls the slow inwards depolarizing current, *k_s* and τ_*s*2_ control the activation gate of the slow inward depolarizing current. The parameters that deviated the least from the baseline BOFC model were τ_*w*2^−^_, $\tau_{w^{+}}$,*k_so_*, and *u*_*s*_. Comparing the parameter sets of GA-derived SNLV and ICMP models, most parameters were significantly different, by 0.4 to 1.9-fold (Figure 2F). The parameters that had the highest fold-change were $\tau_{w^{+}}$ (1.8-fold increase) and τ_*si*_ (1.9-fold increase). These parameters control the inactivating gate of the slow inward current ($\tau_{w^{+}}$), and the slow inward current itself (τ_*si*_), respectively.

### ICMP Models Result a More Pro-arrhythmic Behavior Compared to SNLV Models

**FIGURE 3:**
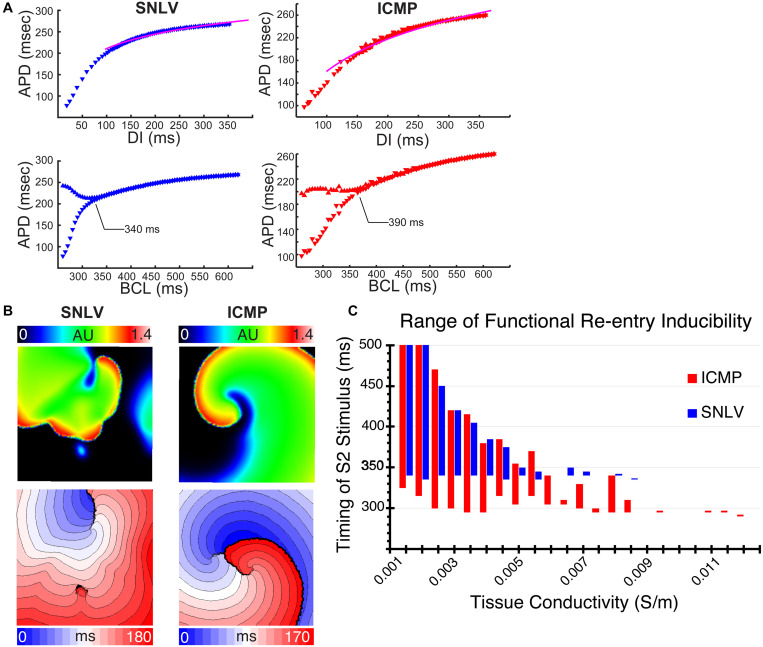
**(A)** Cell-level simulations of the best-fit GA-derived SNLV (blue) and ICMP (red) model. The first row demonstrates the APDR. The pink line is the patient derived average APDR curve. These plots demonstrate good fit of the models to patient data. The second row demonstrates bifurcation plots for SNLV and ICMP models. The onset of APD alternans (development of bifurcation) is annotated. APD alternans occur at slower cycle lengths in the ICMP model compared to SNLV suggesting a more proarrhythmic behavior. **(B)** Tissue-level simulation using a conductivity of 0.009 S/m and S1S2 interval of 330 ms for the SNLV model and 295 ms for the ICMP model. Non-sustained functional re-entry was induced in the SNLV model while sustained functional re-entry was induced in the ICMP. Upper row: snapshot of the simulation, color represents transmembrane voltage in arbitrary units. Lower row: activation isochrone maps for simulations using SNLV and ICMP models; each isochrone represents 10 ms. **(C)** Range of functional re-entry inducibility of the SNLV (blue) and ICMP (red) best-fit models. X-axis represents different tissue conductivity values in Siemens/m and *Y*-axis represents S1S2 coupling intervals that resulted in sustained functional re-entry. The best-fit ICMP model has a wider range of S1–S2 intervals, for all conductivity values, that result in sustained functional re-entry suggesting a more proarrhythmic behavior.

In single cell simulations, GA-derived ICMP models developed APD alternans at a slower pacing cycle length compared to SNLV (390 vs 340 ms, Figure 3A). In both ICMP and SNLV models, APD alternans developed with a fork-type bifurcation and the amplitude of the alternans monotonically increased as the pacing cycle length decreased. Tissue-level simulations had similar APDR with cell-level simulations (Supplementary Figure 3).

In tissue level simulations, the ICMP model was inducible for sustained functional reentry over a wider range of S1S2 coupling intervals over the entire conductivity range, compared to the SNLV model (Figures 3B,C). An example of that is illustrated by Supplementary Videos 1 and 2. Using a conductivity of 0.009 S/m (corresponding to a conduction velocity of 62–64 cm/s) the ICMP model developed sustained functional re-entry at a S1S2 coupling interval of 295 ms (Supplementary Video 1). The single spiral wave that was induced in this case exhibited quasi-stability. For the same S1S2 coupling interval, the SNLV model was not inducible, and it developed non-sustained (<2 s) functional re-entry at an S1S2 of 330 ms (Supplementary Video 2).

### The Population of GA-Derived Models Has Consistent Emergent Proarrhythmic Behavior Across Clusters

The population of SNLV and ICMP action potential models were successfully clustered using AHC. The cophenetic correlation coefficient was 0.97 and 0.98 for SNLV and ICMP, respectively, which suggests excellent hierarchical clustering. We present the clustering dendrograms of the SNLV and ICMP population of GA-derived models in Supplementary Figure 4. Based on this dendrogram analysis we partitioned the population of GA-derived model to the 2 and 4 most dissimilar clusters. Clustering of the GA-derived models was asymmetric, with the vast majority of models clustering in one large cluster (number of GA-derived models corresponding to the highest four branches of the dendrogram was 1506, 3, 2, and 1, for SNLV, and 1506, 4, 1, and 1 for ICMP). Considering the highly skewed distribution of individuals corresponding to the highest four branches of the dendrogram, we further performed sensitivity analysis by constraining the AHC analysis to GA-derived models with parameter values within the 99th and 95th percentile of the entire population of GA-derived models. This allowed us to control for the influence of extreme parameter values in AHC. In sensitivity analysis, when applying AHC to GA-derived models with parameter values within the 99th percentile of the GA-derived model population the number of models corresponding to the highest four branches was: 1283, 4, 2, and 2 (SNLV) and 1265, 5, 2, and 2. For parameter values within the 95th percentile this number was 1005, 78, 26, and 2 (SNLV), and 1120, 63, 49, and 3 (ICMP).

**FIGURE 4:**
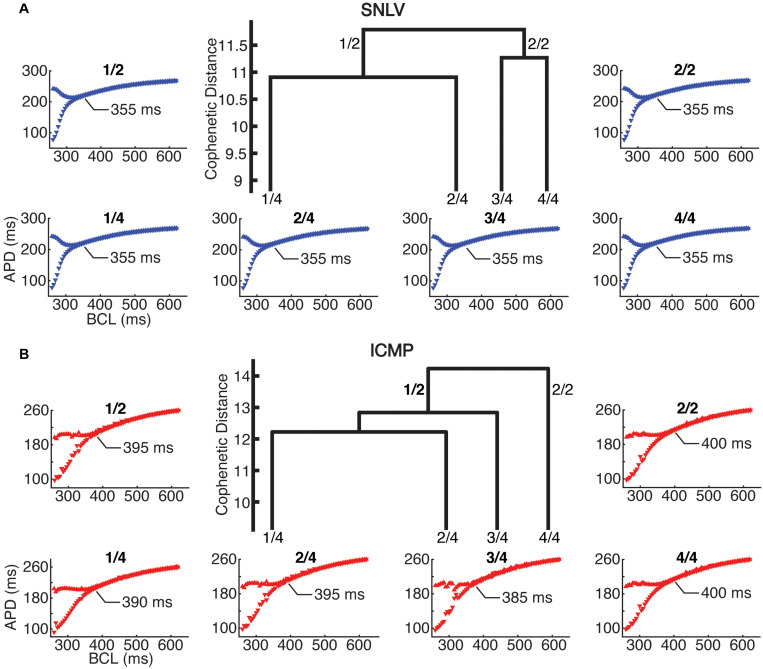
Cell-level simulations in the 2 and 4 highest level clusters of **(A)** SNLV and **(B)** ICMP models. In the center of each sub-plot we show the four highest-level clusters of the dendrogram produced by AHC. The two highest-level clusters are noted as 2/1 and 2/2 and the four highest level clusters are noted as 4/1, 4/2, 4/3, and 4/4. Note that ICMP clusters 2/2 and 4/4 are identical since no bifurcation of the dendrogram occurs at this level. The bifurcation plots surrounding the dendrograms are labeled after the cluster that they have been created from (*X*-axis is pacing cycle length in ms and *Y*-axis is APD in ms). For all clusters, onset of alternans occurred at ICMP models at longer cycle length intervals compared to SNLV models (385–400 ms vs 355 ms).

In cell-level simulations using the parameters at the centroid of the parameter space for the two and four clusters with the highest hierarchy (and most dissimilarity), the proarrhythmic behavior of the models was similar to that of cell- and tissue-level simulations using the best-fit derived model (described in the section above). Specifically, in cell-level simulations, the ICMP models developed APD alternans earlier (385–400 ms) compared to the SNLV models (355 ms, Figure 4).

**FIGURE 5:**
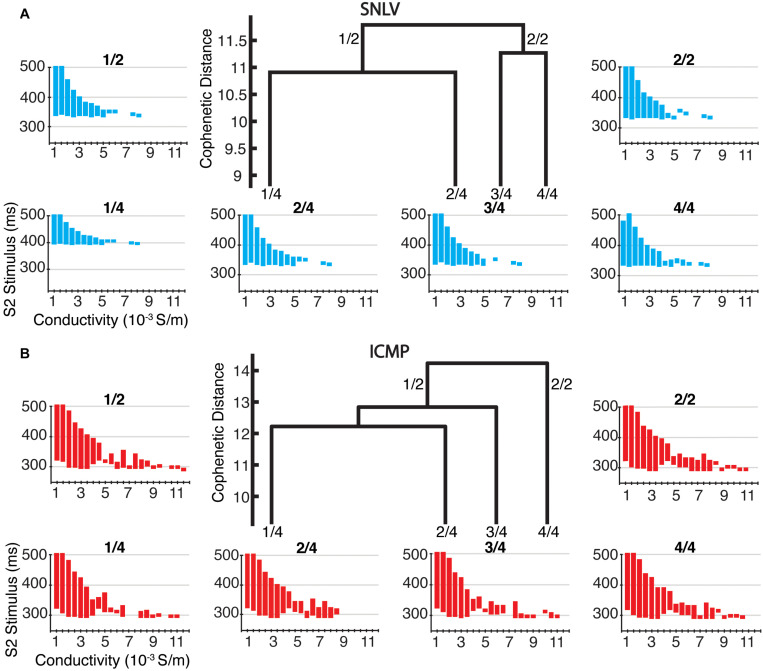
Tissue-level simulations in the two and four highest-level clusters of **(A)** SNLV and **(B)** ICMP models. In the center of each sub-plot we show the four highest-level clusters of the dendrogram produced by AHC. The two highest-level clusters are noted as 2/1 and 2/2 and the four highest clusters are noted as 4/1, 4/2, 4/3, and 4/4. Note that for ICMP clusters 2/2 and 4/4 are identical since no bifurcation of the dendrogram occurs at this level. Each plot surrounding the dendrogram shows the S1–S2 coupling intervals that resulted in sustained functional re-entry for different conductivity values and is labeled after the cluster that it has been created from (*X*-axis represents different tissue conductivity values in mS/m and *Y*-axis represents S1–S2 coupling intervals that resulted in sustained functional re-entry). For all clusters, ICMP models developed sustained functional re-entry over a wider range of S1–S2 coupling intervals for the entire range of conductivity values, compared to SNLV.

In tissue-level simulations the ICMP models developed sustained functional re-entry over a wider range of S1S2 coupling intervals for all conductivity values, compared to SNLV (Figure 5). The results of cell-level and tissue-level simulations using the clusters derived from sensitivity analysis (parameters constrained to the 99th and 95th percentile of their distribution) were similar to those when examining the entire population of GA-derived models.

## Discussion

### Main Findings

The goal of this study was to characterize the electrical restitution properties of the non-infarcted myocardium in ICMP patients, establish a methodology that enables development of action potential models that incorporate cohort-specific APDR, and capture the uncertainty inherent to the process of model development from clinical data. Our main *clinical* finding is that APDR of the non-infarcted LV myocardium of ICMP patients is clinically and statistically significantly steeper compared to SNLV. This suggests that the non-infarcted myocardium of ICMP patients, despite having normal (>1.5 mV) bipolar voltage amplitude on EGMs, is remodeled and has different EP properties from SNLV. The increased within-subject variance of APDR slope in ICMP patients suggests heterogeneous substrate with respect to repolarization dynamics. Furthermore, the magnitude and statistical significance of the difference of APDR slope between ICMP and SNLV was robust in sensitivity analysis and independent of the PVC/VT status of the patients. This suggests that the underlying disease process and cardiac remodeling is what determines APDR, rather than the presenting arrhythmic phenotype.

Our main *in silico* finding is that GA-derived ICMP models exhibited a more pro-arrhythmic behavior in cell-level and tissue-level simulations compared to SNLV. This is the first study to show that EP measurements performed with equipment routinely available in the electrophysiology laboratory can be used to develop action potential models. The parameters of the GA-derived ICMP models were up to 1.9-fold different compared to SNLV. The steeper APDR slope present in ICMP has a critical effect in tissue-level emergent EP behavior, promoting the development of sustained functional re-entry. Although conduction velocity was not assessed in the patient cohort, we performed tissue-level analysis over a wide range of conductivity values making our results generalizable to different tissue conductivity states. These results highlight the importance of developing action potential models using clinically derived EP properties such as APDR, since generic action potential models do not capture clinically assessed EP properties of healthy or diseased myocardium.

A novel finding is that despite the variability present in the population of GA-derived models, GA-derived models can be clustered in a hierarchical cluster tree and the emergent pro-arrhythmic behavior of the highest-level (most dissimilar) clusters are similar in cell and tissue-level simulations. This suggests that: (a) there is significant redundancy built in the GA-derived models; and (b) our model development approach was reliable with small variability and uncertainty in the population of derived models. Despite variability in individual parameter values and the exact results produced by simulations using different GA-derived models, the emergent behaviors that are relevant in arrhythmogenesis were preserved.

### Comparison With Results of Other Clinical Studies Assessing APDR

There are no prior studies comparing APDR of non-infarcted LV myocardium of ICMP patients to SNLV. Our results are consistent with what has been previously reported in studies assessing APDR of the RV myocardium in patients with SHD. Our ARI measurements in the LV of ICMP patients are similar to what has been previously reported using monophasic action potential (MAP) catheters in the RV of patients with SHD (274 ± 42 ms for 600 ms, 258 ± 35 ms for 500 ms, 237 ± 29 ms for 400 ms, and 219 ± 24 ms for 330 ms, *n* = 42) (Selvaraj et al., 2007; Dorenkamp et al., 2013). Koller et al. (2005) using MAP catheters, evaluated APDR in SHD patients (*n* = 24) and SNLV (*n* = 12). Similar to our results, there was no difference in baseline APD between SNLV and SHD patients, when pacing at 600 ms. The reported baseline APD was similar to ours (277 ± 5 ms for SHD and pacing cycle length of 600 ms). However, in our study the restitution curves of SNLV and ICMP separated at longer DIs compared to Koller et al. (2005). Our SNLV cohort had tighter confidence intervals for both DI and ARI. Furthermore, we demonstrated a robust and statistically significant difference in APDR slopes between patients with SNLV and ICMP, while the study of Koller et al. (2005) failed to show that. This may be due to: (a) our SNLV cohort was more homogeneous compared to that of Koller et al. (2005); (b) patients with non-ICMP may have different APDR properties compared to ICMP and Koller et al. (2005) analyzed both groups combined; and (c) RV septal APDR may be affected differently compared to LV myocardium in ICMP.

### Mechanistic Insights of APDR Dynamics in ICMP

The steeper APDR observed in patients with ICMP can be attributed to changes in the autonomic nervous tone and/or electrophysiologic remodeling present in patients with ICMP. Although the scope and design of this study do not allow for conclusions regarding the cellular mechanisms underlying the steeper APDR curves observed in ICMP, our findings can be hypothesis generating. The steeper APDR described in patients with ICMP is consistent with findings in patients with heart failure (Koller et al., 2005; Selvaraj et al., 2007; Dorenkamp et al., 2013), rather than experimental models of acute ischemia (Dilly and Lab, 1988). Patients with heart failure have augmented sympathetic tone (Florea and Cohn, 2014). Activation of the sympathetic system resulted in increased steepness of the APDR slope in a human in vivo study using MAP recordings from the right ventricle and isoprenaline or adrenaline administration (*n* = 18) (Taggart et al., 2003). Although the electrophysiological remodeling in humans with heart failure (including ICMP) remains to be elucidated, limited data suggest: (a) decrease in delayed rectifying K^+^ currents (Beuckelmann et al., 1993); (b) activation of late Na+ currents (Horvath and Bers, 2014); and (c) increase in intracellular Ca^++^ and Ca^++^ transients in heart failure (Gorski et al., 2015). Experimental data from animal studies support that all these changes can result in steeper APDR: (a) inhibition of I Ks using chromanol 293B resulted in a steeper APDR curve in a swine myocardial tissue study (Jing et al., 2014); (b) selective inhibition of late sodium currents with GS967 resulted in flattening of the APDR in Langendorff-perfused rat hearts (Pezhouman et al., 2014); and (c) Suppression of Ca++ transients with thapsigargin and ryanodine resulted in flattening of the maximum APDR slope in patch-clamped rabbit ventricular myocytes (Goldhaber et al., 2005).

### Comparison With Other Studies of Model Fitting to Clinically Assessed APDR

There are no other studies to date that have fitted action potential models (other than the Mitchel-Shaffer model) to APDR of ventricular myocardium of SNLV and ICMP patients. We will not discuss fitting of the Mitchel-Schaffer model (Relan et al., 2011), since in this model APDR slope can be directly calculated from model parameters (Mitchell and Schaeffer, 2003), thus its model fitting methods are not applicable to any other action potential model. GAs have been previously used to fit action potential models to experimental measurements (Syed et al., 2005; Bot et al., 2012; Kaur et al., 2014; Groenendaal et al., 2015; Cairns et al., 2017; Devenyi et al., 2017), but this is the first study to use GA on clinically obtained APDR. Lombardo et al. (2016) fitted the Fenton-Karma and Koivumäki atrial models to patient-specific APDR, action potential shape (obtained with MAP catheters), and conduction velocity restitution from patients with atrial fibrillation. They used simulated annealing for model derivation. They demonstrated that patient-specific models exhibit different dynamics compared to baseline models. In our model derivation algorithm, we did not incorporate a clinically derived action potential morphology or conduction velocity restitution because they cannot be reliably assessed with clinically available methods. We did not want to constrain our GA to measurements with high experimental uncertainty: (a) MAP catheters have not been validated to capture the waveform of the cellular action potential and in a prior study MAP-derived waveforms have different morphology compared to true trans-membrane potential waveforms (Kondo et al., 2004); (b) accurate estimation of conduction velocity requires construction of high-resolution isochrones and patient-specific geometries (Cantwell et al., 2015). Contrary to our study, Lombardo et al. (2016) did not have a control group to compare the emergent dynamics between healthy and diseased. Last, Lombardo et al. (2016) report the dynamics of the “best-fit” derived model, and they do not assess for the uncertainty in the population of derived models yielded by simulated annealing.

### Significance for Virtual Heart Modeling

Our study helps overcome critical barriers to incorporating the EP remodeling of ICMP patients in cardiac action potential models. First, there is a paucity of studies assessing the APDR of the non-infarcted LV myocardium in ICMP patients. In this study we provide absolute values of APD and APDR slope of the non-infarcted myocardium of the LV of ICMP patients. We also demonstrate a wide within- and between-subject variance in APD/APDR values of the non-infarcted LV myocardium in ICMP patients. This variance reflects the heterogeneous substrate in ICMP patients and can be used in variability and uncertainty quantification studies of organ-scale simulations performed for VT localization and ablation planning in ICMP patients.

Second, there are no established methods for incorporating clinically assessed APDR in models of cardiac EP. Previous clinical studies have adapted the Mitchell-Schaffer model to reproduce clinically assessed APDR by either manually adjusting model parameters (Keldermann et al., 2008; Chen et al., 2016), or by using deterministic optimization methods (Relan et al., 2011). However, the Mitchell-Schaffer model is unique in having an algebraic expression of its APDR slope directly derived from the model parameters (Mitchell and Schaeffer, 2003) and thus these methods cannot be applied to any other action potential model. In this study, we provide a computational framework and pipeline that enables development of cardiac action potential models to clinically assessed APDR. We demonstrated that GA-derived models capture the disease-specific pro-arrhythmic phenotype. Whole heart simulations using GA-derived action potential models have the potential to be more accurate in predicting the risk and location of VTs, as well as to predict the transition of VT to VF, but this needs to be tested in future studies. Although our study focuses on ICMP and uses the BOFC as a baseline model, the pipeline that we developed can be used in any myocardial disease that affects ventricular APDR and any phenomenological or biophysically detailed cardiac action potential model.

Last, there is a significant uncertainty associated with the process of model development form experimental data. This stems from the facts that: (1) there is uncertainty with respect to variability in experimental measurements; and (2) optimization problems involving non-linear differential equations do not yield unique solutions. Stochastic optimization approaches have been used in pre-clinical studies to derive populations of models that reproduce experimental APDR (Groenendaal et al., 2015; Cairns et al., 2017; Devenyi et al., 2017). There is considerable variability in the model dynamics amongst different models yielded by this approach (Cairns et al., 2017), and there have been no studies examining for common emergent behaviors amongst these parameters. As described in the following section, with this study we couple machine learning with computer-based simulations to establish a pipeline of action potential model development that accounts for the uncertainty related to the process of model development from clinical data.

### Significance for Clinical Risk Stratification Using Virtual Heart Modeling

There are no studies assessing whether the invasively acquired slope of the APDR is related to the risk for hard clinical outcomes such as the onset of ventricular arrhythmias and cardiac death. Previous studies assessing ECG-derived APDR surrogates report increased risk for ventricular arrhythmias or death in patients with ICMP (Nicolson et al., 2014) and non-ICMP (Nicolson et al., 2021) who have steep restitution slopes. However, the hazard ratios reported are 4–4.1 and the ROC 0.61, suggesting that restitution slope alone has at best, modest predictive value. In addition to APDR slope, APDR slope spatial dispersion is critical for arrhythmogenesis. Virtual Heart modeling that incorporates patient and site specific APDR has the potential to be a superior risk prediction tool to static biomarkers such as the APDR slope, and other well-established risk predictors of ventricular arrhythmias or death such as LV ejection fraction and myocardial fibrosis. In a previous study by our group, computer-based simulations in patients with ICMP significantly overperform well-established biomarkers such as left ventricular ejection fraction and myocardial fibrosis on MRI (Arevalo et al., 2016). This study indicates that Virtual Heart modeling that has as an input patient-specific distribution of fibrosis, yields a significantly higher predictive value compared to fibrosis itself. Virtual Heart modeling simulations that incorporate patient-specific distribution of APDR have the potential to yield a higher predictive value compared to APDR itself, but this needs to be examined in future studies.

In this study we developed action potential models using average, cohort-specific, clinically obtained APDR, but this pipeline could be used for development of action potential models to patient-specific APDR and even region-specific APDR (within the same patient), capturing the spatial dispersion of APDR. APDR heterogeneity is a critical substrate for arrhythmogenesis and degeneration of VT to VF (Banville and Gray, 2002; Keldermann et al., 2008) and it is not currently incorporated in virtual heart models. Currently, having patient-specific or region-specific APDR information is not clinically feasible without an invasive EP procedure. However, with advances in electrocardiographic imaging (Cluitmans et al., 2018), non-invasive imaging of repolarization may be feasible in the near future. Clinical assessment of APDR would then be possible to be performed non-invasively during non-invasive programmed electrical stimulation. The framework that we present here could be used to develop virtual heart models from non-invasively acquired APDR data. However, further optimization of the methodology that we present here would be needed for such a task, since it is computationally intense.

### Significance of Establishing a Methodology That Captures the Uncertainty Inherent to Action Potential Model Development

Coupling machine learning with computer-based simulations is an emerging approach as it provides means to comprehensively analyze the wealth of high-dimensional, complex data produced by simulations (Cantwell et al., 2019). In this work we demonstrate a novel application of coupling machine learning with computer-based simulations. We show that machine learning can be used to effectively summarize and cluster the population of models derived from a stochastic optimization method. In our study, similar to other studies (Groenendaal et al., 2015; Cairns et al., 2017; Devenyi et al., 2017; Krogh-Madsen et al., 2017), there is considerable variability in the population of GA-derived models. Reporting the dynamics of the “best-fit” GA-derived model is not adequate since a unique “best-fit” model does not truly exist. AHC allows for a simple visualization of the distance and relationship between different parameter sets in the entire population of derived models, effectively organizing the space of derived models into distinct clusters. The GA-derived model clusters that are most dissimilar can be identified using dendrograms and the cophenetic distance metric. Simulations can be executed with the parameter space centroids of these clusters and the results of these simulations can be compared to assess for differences in model dynamics.

This is important because simulations using the parameter space centroids of clusters produce the average dynamics of the population of derived models rather than the dynamics of the non-unique “best-fit” model. If the emergent dynamics of interest are the same between the best-fit model and the most dissimilar clusters, this suggests a reliable model development with small variability and uncertainty in the population of derived models. Alternatively, if the emergent dynamics of interest are different between the best-fit derived models and/or different clusters, this suggests that either the model derivation method was inadequate or that there is more than one emergent dynamic behavior in the derived model population. If there are concerns of inadequate model development, then a more detailed sensitivity analysis and tuning of the model development algorithm is warranted. Otherwise, the presence of more than one emergent dynamic behaviors among different clusters demonstrates the variability and uncertainty present in the derived model population with respect to the emergent behaviors of interest. Should such derived models be used in Virtual Heart modeling studies for different hypothesis testing, then simulations should be executed using the centroids of clusters with different emergent behaviors. This will result in the uncertainty present in the population of derived models to be captured in the Virtual Heart modeling study.

### Limitations

This study has several limitations. First, we developed action potential models using cell-level simulations, whereas the clinical measurements represent a tissue-level behavior. Development of action potential models using tissue-level simulations might yield different parameter results. However, considering the large population of individuals and generations that we used in the GA, model development using tissue-level simulations would be computationally not tractable. To address this limitation, we performed tissue-level simulations to assess the APDR of GA-derived models. Both for SNLV and ICMP models, APDR in tissue-level was similar to that of cell-level simulations. Second, we did not incorporate action potential morphology specific to SNLV and ICMP patients in the model development process. We rather used the same action potential morphology template, derived from the baseline BOFC model, to constrain the model development process (see Supplementary Materials and Methods). Incorporating different action potential morphologies may have resulted in different parameter estimates. Currently there are no clinically available methods to accurately assess the action potential morphology. Third, the differences in SNLV and ICMP model parameters do not necessarily correspond to *in-vivo* difference in cellular EP. Phenomena such as post-repolarization refractoriness cannot be explicitly captured. Development of action potential models that capture true cellular EP would require cellular EP measurements and maneuvers (Groenendaal et al., 2015; Devenyi et al., 2017) that were outside the scope of this study. Last, model development using the approach that we present here is computationally intense. This precluded us from developing multiple patient- and site-specific models that could then subsequently used in tissue level simulations that would incorporate heterogeneity. The focus of this study was to demonstrate feasibility and not to optimize performance. Fewer iterations from those that we performed in our GA may be adequate to obtain models that capture the pro-arrhythmic behavior of the substrate, but this needs to be tested in future studies.

## Conclusion

This is the first study to characterize the electrical restitution properties of the non-infarcted LV myocardium of ICMP patients. We clinically characterized the pro-arrhythmic substrate of ICMP patients and demonstrated that it has a steeper APDR compared to SNLV, indicating the presence of EP remodeling in the non-infarcted LV myocardium. *In silico*, we demonstrated that ICMP APDR contributes to a more pro-arrhythmic tissue-level behavior. We coupled virtual heart modeling with machine learning to establish a robust and reproducible methodology to incorporate easily obtainable clinical EP measurements to cardiac action potential models and capture the uncertainty inherent to the model development process. We demonstrated that action potential models derived from ICMP patients capture the pro-arrhythmic potential of the underlying disease in simulations. Importantly, we showed that despite the variability in the derived action potential model population, even the most dissimilar clusters formed by this population exhibit the same emergent pro-arrhythmic behavior in cell- and tissue-level simulations. The methods that we present here can be used for model development in any disease state that affects ventricular repolarization and restitution, contributing to the emergent field of precision medicine. Virtual heart models incorporating disease-specific EP properties have the potential to result in improved risk stratification and therapeutic planning and this is the focus of our future research.

## Data Availability Statement

The raw data supporting the conclusions of this article will be made available by the authors, without undue reservation.

## Ethics Statement

The studies involving human participants were reviewed and approved by the Johns Hopkins IRB. The patients/participants provided their written informed consent to participate in this study.

## Author Contributions

KA did substantial contributions to the conception and design of the work, the acquisition, analysis and interpretation of the data for the work, and drafting the work. RB, PB, HA, and NT did substantial contributions to the design of the work and data interpretation and revising it critically for important intellectual content. TB, JC, SJ, JM, SS, HT, and HA did substantial contribution to data acquisition, analysis, and interpretation. All authors provided the approval for publication of the content and agreed to be accountable for all aspects of the work in ensuring that questions related to the accuracy or integrity of any part of the work were appropriately investigated and resolved.

## Conflict of Interest

The authors declare that the research was conducted in the absence of any commercial or financial relationships that could be construed as a potential conflict of interest.

## Abbreviations

AHC: agglomerative hierarchical clustering
APD: action potential duration
APDR: action potential duration restitution
ARI: activation-recovery interval
BOFC: Bueno-Orovio-Fenton-Cherry (action potential model)
DI: diastolic interval
dV/dt: first derivative of voltage with respect to time
EP: electrophysiological
GA: genetic algorithm
ICMP: ischemic cardiomyopathy
LV: left ventricle
PVC: premature ventricular complex
RV: right ventricle
SHD: structural heart disease
SNLV: structurally normal left ventricle
UniEGMs: unipolar electrograms
VF: ventricular fibrillation
VT: ventricular tachycardia.
