# Supplementary material for: Characterization of the Electrophysiologic Remodeling of Patients With Ischemic Cardiomyopathy by Clinical Measurements and Computer Simulations Coupled With Machine Learning
Source: Front Physiol. 2021 Jul 14;12:684149. doi: 10.3389/fphys.2021.684149 (PMC8317643; doi:10.3389/fphys.2021.684149)
Supplement: Supplementary file 1 [file Data_Sheet_1.PDF]

SUPPLEMENTAL MATERIAL for

Characterization of the electrophysiologic remodeling of patients with ischemic cardiomyopathy by clinical measurements and computer simulations coupled with machine learning

Konstantinos N. Aronis, MD*; Adityo Prakosa, PhD; Bergamaschi T, Ronald Berger, MD, PhD; Patrick M Boyle, PhD; Jonathan Chrispin, MD; Suyeon Ju; Joseph Marine, MD; Sunil Sinha, MD; Hari Tandri, MD; Hiroshi Ashikaga, MD, PhD; Natalia A Trayanova, PhD.

*To whom correspondence should be addressed.

E-mail: karonis1@jhmi.edu

Supplemental Material and Methods

1. *Signal processing for ARI derivation*

To extract the ARI, we used the Uni EGM signal. First, we aligned all beats to the pacemaker artifact (Supplemental Figure 1A). We detected the pacemaker artifact using a high-pass filter at 170 Hz [Parks-McClellan, finite impulse response (FIR) filter with passband frequency at 170 Hz, stopband frequency at 190 Hz, maximum passband ripple 1dB, and stopband attenuation 80dB] and a peak detection algorithm. Second, we found the activation time (AT) of each beat defined as the time of the minimum first derivative of voltage (*V*) with respect to time (*dV/dt*) of the UniEGM ventricular depolarization waveform in a search window of 40 msec after the onset of the UniEGM. We manually selected the onset of ventricular depolarization on a "butterfly plot" where all beats where overlaid. To control for experimental noise and improve the reproducibility of minimum *dV/dt* selection, we filtered the *dV/dt* signal of the UniEGM using a low-pass filter at 80 Hz (minimum-order equiripple FIR filter with passband frequency 80 Hz, stopband frequency 110 Hz, maximum passband ripple 1dB, and stopband attenuation 80 dB). Third, we aligned all beats to the activation time (Supplemental Figure 1B). We signal averaged the aligned beats. Fourth, we found the recovery time (RT) of the signal-averaged beat defined as the maximum *dV/dt* of the ventricular repolarization UniEGM waveform in a search window between the end of the ventricular activation UniEGM component and the beginning of the next beat. We selected this search window manually. To decrease signal noise and improve the reproducibility of minimum *dV/dt* selection, we filtered the *dV/dt* signal of each UniEGM using a low-pass filter at 30 Hz (minimal-order equiripple FIR filter with passband frequency at 30 Hz, stopband frequency at 60 Hz, maximum passband ripple 1dB, and stopband attenuation 80dB), before signal-averaging the UniEGM signals. Application of low-pass filters at 30 Hz have been recommended for detection of ARI in previous studies, since it decreases noise and improves the reliability of the ARI measurement.[1] Last, we calculated the time interval between activation and recovery times. This interval is the ARI of the signal-averaged beat. This signal-averaged beat was then used as the template in the template-matching algorithm used to find the ARI of each individual beat, as described in the next section.

2. *Template matching algorithm for ARI derivation*

To find the ARI of each individual beat we performed template matching of each beat with the signal-averaged beat. Template matching algorithms have been used in ECG signal processing to provide reproducible measurements of repolarization indices, considering the temporal liability in ventricular repolarization.[2, 3] Here we adapted a template matching method initially developed by Berger et al. for assessment of QT variability.[2, 3] Specifically, for each beat (*n*), we derived a time stretching (or squeezing) factor F_n such that, F_n minimizes the sum-squared error between the waveform of the *dV/dt* of beat *n* and the *dV/dt* of the signal-averaged

beat (Supplemental Figure 1C). The waveform that is included in this calculation is the UniEGM *dV/dt* from the end of the ventricular depolarization component of the UniEGM until the next beat. It is the same waveform that we used for calculation of RT in the signal averaged beat in the section above. Specifically, F_n is defined as:

$$F_n = \underset{F_n}{\operatorname{argmin}} \sum_{j=1}^{T} [\phi(j) - x_n(F_n \cdot j)]^2 \quad (1)$$

Here $\phi(i)$ is the *dV/dt* of the signal-averaged waveform, $x_n(i)$ is the *dV/dt* of the waveform of each individual beat *n* and *i* is the sample number. F_n is the time stretching factor for beat *n*. Summation is over each sample *j* of the RT search window (from sample 1 to the last sample *T*). We solved this minimization problem using a 3-step grid search method. In the first step the value F^1_n that minimizes (1) was found by searching the interval 0.5 to 1.5 with a step of 0.01. In the second step, the value F^2_n that minimizes (1) was found by searching the interval $F^1_n - 0.02$ to $F^1_n + 0.02$ with a step of 10^{-4}. In the last step, the value F^3_n that minimizes (1) was found by searching the interval $F^2_n - 2 \cdot 10^{-4}$ to $F^2_n + 2 \cdot 10^{-4}$ with a step of 10^{-6}. The value F^3_n is the stretching factor F_n of beat n.

To find the ARI of each beat, we multiplied the ARI of the signal-averaged beat with the stretching factor F_n of each beat:

$$ARI_n = F_n \cdot ARI_{template} \quad (2)$$

Here ARI_n is the ARI of each beat (*n*) and F_n is the stretching factor of beat *n*.

3. *Curve fitting for derivation of APDR intercepts and slopes from calculated ARIs*

To derive APDRs from the calculated ARIs, we first plotted the ARI of each beat against the previous diastolic interval. We excluded the first 3 beats and the last beat of the burst pacing sequence from the analysis, to allow the UniEGM morphology to be at steady-state. We considered beats that had an ARI > 75% of the signal-averaged beat ARI at any pacing cycle length as outliers, and we excluded them from analysis. We performed a bi-square regression analysis performed to fit a logarithmic function of the form:

$$ARI_n = b_0 + b_1 \ln(DI_{n-1}) \quad (3)$$

Here ARI denotes the activation recovery interval of the nth beat, DI the diastolic interval of the previous beat (*n-1*), b_0 is the intercept of the restitution curve with the x (DI) axis, b_1 is the slope of the restitution curve and *ln* denotes the natural logarithm function. (Supplemental Figure 1D). We performed bi-square regression as it is robust to heteroskedasticity and outliers that are inherent to our data.[4, 5] For each curve we calculated the coefficient of determination R^2. Curves with a coefficient of

determination < 50% were excluded by the analysis as they represent poor quality signals.

4. Use of genetic algorithms for development of action potential models

4.1. Baseline model used in our genetic algorithms

To proceed with action potential model development from the clinical data, we needed a starting action potential model with small number of variables and parameters that would allow for computationally tractable execution of the genetic algorithm. The Bueno-Orovio, Cherry, Fenton (BOFC) is a phenomenological action potential and wave propagation model. It reproduces important tissue-level characteristics of epicardial, endocardial and midmyocardial cells, including action potential amplitude, and morphology, upstroke velocity, steady-state action potential duration (APD) and conduction velocity restitution, minimum APD, and minimum diastolic interval. The BOFC model has 4 state variables, 3 ionic currents, and 28 parameters. This model has minimal memory and can reach quasi-steady state within 10 beats.[6] The simplicity of this model allows for fast computation and fitting using stochastic optimization approaches, where a large number of iterations is required. A complete description of the model can be found in the original paper by Bueno-Orovio et al.[7] For the purposes of our study we used the endocardial parameter set of the BOFC model, as the baseline model. These parameters are presented in Supplemental Table 4. The reason for this was that all of our measurements of ARI and APDR was from the endocardial aspect of the left ventricle. The bifurcation plot of the baseline model in single cell simulations and its range of functional re-entry inducibility in tissue level simulations are shown in Supplemental Figurer 10 A and B.

4.2. Design and implementation of the genetic algorithms

To fit the baseline model to the average APDR curves derived from ICMP patients and SNLV, we used a biologically-inspired meta-heuristic known as genetic algorithm (GA). GA is stochastic optimization approach that mathematically simulates the three steps of evolution: natural selection, recombination and mutation (Supplemental Figure 2). We fitted 15 of the 28 parameters of the baseline model. We based the parameter selection on a sensitivity analysis and previously published literature. We discuss parameter selection in detail in the sub-section “sensitivity analysis and parameter selection” below. The way that we designed and implemented the GA is as follows:

We started the GA with a population of 1,512 randomly-selected 15-dimensional vectors. Each vector constitutes an “individual” for the purposes of the GA and contains a randomly selected set of model parameters. We created the initial population by uniform sampling of the parameter space with lower and upper bounds[7] as specified in Supplemental Table 5. Similar to the approach by

Groenendaal et al,[8] we run 10 different GAs using different initial populations. The results of these 10 GA runs were combined into a final GA run. In this final GA run, the initial population was drawn from a parameter space that bounds the solutions obtained from the 10 initial GA runs (Supplemental Tables 2&3). The parameter space used in the last GA step is shrunk compared to the initial parameter space used in the 10 different runs. This approach is known as the "local iterative" approach and is associated with better accuracy of GAs.[8, 9]

We then assigned a "fitness score" to each individual parameter set. To calculate the "fitness score", we first defined an objective function that quantifies the error between the APDR derived from a cell-level simulation using an individual parameter set, and the clinically determined APDR in SNLV or ICMP. We provide more details on the objective function in the sub-section "objective function" below. We then scaled the *Error* value that is returned by the objective function to produce the "fitness score" of each individual. The scaling was performed using the rank approach. With this approach we assigned a rank *r* to the objective function results (i.e. 1 to the lowest *Error*, 2 to the second lowest *Error* etc..). The "fitness score" of an individual with rank *r* is $fitness = 1/\sqrt{r}$. We then selected parents using a tournament selection function of size 4. This selection function randomly chooses 4 potential parents and then it selects the best individual of these 4 candidates to be a parent for the next generation.

We created the next generation of individuals by recombination (cross-over) and random alternations (mutation) of individuals selected as parents. We created 80% of the individuals of the next generation by cross-over and 20% by mutation. We based our decision to use 80% cross-over rate on the analysis that we present in the sub-section "cross-over rate". We performed cross-over using the "scattered cross-over" function. This function selects two parents randomly. It then generates a 15-dimensional vector (i.e. the same dimension with the parameter vector of the parent), that consists of randomly-selected 1s and 0s. Elements that have been assigned 1 are chosen from the first parent and elements that have been assigned 0 will be chosen from the second parent. We introduced mutations using the adaptive-feasible mutation function. The adaptive feasible mutation function randomly generates mutations that are adaptive with respect to the last successful or unsuccessful generation and within the defined bounds of the parameter space. Introduction of mutations protects the GA from getting stuck in a premature local minimum. In our GA we also included elitism with an elite count of 2. Elitism with a count of 2 means that the 2 best fit individuals of each generation will not cross with any other individuals and will not get mutated but rather they will be carried to the next generation unaltered. Elitism facilitates preservation of individuals with very good fitness in the subsequent generations.

We iteratively repeated the steps of (a) evaluating the fitness of individuals, (b) selecting parents from one generation and (c) producing the next generation with

recombination and mutation, until the GA convergences or runs for 100 generations, whatever comes earlier. We defined convergence if for 50 consecutive generations the population fitness did not improve more than 10^{-6}. For the final optimization step, we defined the maximal allowed generation number to be 300 instead of 100. This optimization problem was constrained within the parameter space defined by the bounds in Supplemental Table 5 (for the 10 first GA runs) and in Supplemental Tables 2&3 (for the final GA run). The result of the GA is a population of 1,512 parameter sets. In our analysis we used not only the best fit GA-derived model, but also explored the parameter space of the entire population of GA-derived models using an unsupervised machine learning approach that we describe in the material and methods section of the main manuscript.

4.3. Sensitivity analysis and parameter selection

We selected the parameters of the baseline model that we included in the GA following the approach proposed by Groenendaal et al.[8] Out of the 28 model parameters, we kept constant the u_o parameter (resting membrane potential of 0). For the remaining 27 parameters we performed a series of tests to decide on whether each parameter should be included or the GA. Specifically, we assessed for (a) the effect of parameter variations on APDR, (b) parameter convergence, (c) the parameter distribution in the top 10% fittest individuals, (d) convergence of the parameter variance, and (e) the presence of cross-correlation between parameters.

To assess the effect of parameter variation on APDR (a), we varied each parameter by 20-fold, (on a logarithmic scale, 20 equidistant fold-increase steps and 20 equidistant fold decrease steps), and executed cell-level simulations using the dynamic APDR burst pacing protocol described in the “cell-level simulations” section of the main manuscript. We evaluated how changes in the parameter value affect the APDR. Parameters that minimally only affected APDR were excluded from the analysis (Supplemental Figure 5).

To assess test items (b)-(e) mentioned above we run our GA in “model recovery” mode. Specifically, we run the GA as specified in the section “Design and implementation of genetic algorithm”, but we fitted the model parameters to the restitution curve of the baseline model. We run the GA 12 times, 4 times each with the following settings: (a) initial population of 1512, maximal number of generations 100, (b) initial population of 2508, maximal number of generations 60, and (c) initial population of 504, maximal number of generations 300. If the parameter would fail on average any of the tests (b)-(e) it would be excluded from the analysis.

To assess for parameter convergence (b), we plotted the parameter value of each individual in the population as it evolves over generations (Supplemental Figure 6). We constructed frequency histograms of the parameter’s value in the first and last generation of the GA. If the parameter space would shrink > 50% by the end of the GA run, the parameter would pass this test.

To assess the parameter distribution in the 10% fittest individuals (c) against the remaining individuals, we examined the 10th generation of the GA. We divided the 10th generation in the 10% fittest individuals and the remaining individuals. We plotted the distribution of the parameter values in the two groups using a notched box plot. We compared the parameter distributions statistically using the Kolmogorov-Smirnov test. If the parameter distribution was significantly different in the 10% fittest individuals compared to the remaining individuals, the parameter would pass this test (Supplemental Figure 7A).

To assess for convergence of the parameter variance (d) we plotted the parameter's variance, normalized by its mean-squared error at each generation. We calculated the parameter's variance and mean-squared error from all individuals in each generation. If the normalized parameter variance would monotonically decrease to a minimal value and then plateau, the parameter would pass this test (Supplemental Figure 7B).

Last, to assess for cross-correlation (e) between different parameters, we performed pairwise correlation analysis between all parameter sets in the individuals of the 10th generation of the GA. If two parameters would have a calculated Pearson's correlation coefficient > 0.5 (with a p-value <0.05) one of the two parameters would be excluded from the analysis (Supplemental Figure 8). In these cases, we excluded the parameter that had performed, on average, worse at the tests (b)-(d) described above.

With this approach we excluded 12 parameters from the analysis. In addition, we exclude the parameter u_u, although it did not meet the exclusion criteria described above. U_u represents the peak action potential voltage and has been previously held constant in other studies of optimization.[7] The parameters that were included in the GA are listed in Supplemental Table 5.

4.4. Objective function

The objective function that used in our GA was:

$$Error = 100 \cdot RMSE + MAE \quad (4)$$

Here, for each individual parameter set evaluated by the objective function, *Error* is the total error returned by the objective function. *RMSE* is the root mean square error of the action potential shape derived from a cell-level simulation of an individual parameter set against a template action potential. We included a template action potential in the objective function to serve as a constrain to the optimization problem, limiting the potential GA-derived models to those producing a realistic action potential waveform. The template beat was a single beat at 600 msec, derived from the baseline model. We discretized the action potential template at 1 ms. We selected the scaling factor of 100 in equation 4 above, after performing runs of the

genetic algorithm using values ranging from 25-200 with an increment of 25, and we selected a scaling factor that would result in an equal contribution of RMSE and MAE in the objective function.

MAE is the mean absolute error of the APDR of an individual parameter set against the APDR derived from either SNLV or ICMP. To create the APDR curve for each individual parameter set we executed single-cell simulation using a decremental burst pacing protocol from 600 to 300 ms and a step of 25 ms. Each burst consisted of 10 beats. We analyzed the last 2 beats of each burst to ensure steady-state has been achieved. Beats and/or cycle lengths that resulted in 2:1 block or higher were excluded from the analysis. We defined APD_{sim} at the 10% of the peak action potential voltage (APD90). We extracted the DI_{sim} of the last two beats of each burst. We calculated *MAE* by using only DI_{sim}s that were within the range of clinically assessed DIs. We used the selected DI_{sim}s in the APDR function derived from SNLV or ICMP patients to yield the predicted cohort-specific APD_{pred}. The mean absolute difference between APD_{sim} and APD_{pred} was the *MAE* that we used in the objective function.

4.5. Cross-over rate

To choose the cross-over rate we run the GA for 25 iterations using the APDR of SNLV as the optimization goal (arbitrarily selected). We varied the cross-over rate from 0 to 100% with an increment of 10%. We plotted the mean error (*Error*) of the population over 25 generations and the mean error and 95% confidence interval of the last generation for each cross-over rate (Supplemental Figure 9). We chose the cross-over rate that was associated with the minimal error at the 25th generation. In our analysis the cross-over rate was associated with the minimal error at the 25th generation was 80%.

5. Numerical methods used for simulations

We performed cell-level simulations using the finite difference method and the explicit Euler scheme for all ordinary differential equations of the derived ICMP and SNLV models, with an integration time step of 25 μs. The same approach was used for single-cell simulations of the baseline model used in the GA optimization process. For tissue-level simulations we used the finite element method and the Crank–Nicolson scheme. We discretized the 2 cm x 2 cm x 0.25 mm slab to 50,966 tetrahedral elements. These tetrahedral elements had a mean edge length of 271.58 μm (range 146.74-411.80 μm), and aspect ratio ≤ 2.45. We performed numerical integration in time with a time step of 50 μs. We used Neuman boundary conditions in tissue-level simulations. We used a look up table for calculation of the voltage state variable *u* both in cell-level and tissue-level simulations. Numerical integration was performed using the CARP software for computational electrophysiology (CAPR; Johns Hopkins University, University of Bordeaux, University of Graz),[10] on a parallel computing system.

6. *AHC machine learning method for classification of the populations of GA-derived models.*

In our analysis, we first calculated the distance between every pair of parameter sets in the population of GA-derived models, using the standardized Euclidian distance metric (in a 15-dimensional space). We normalized each parameter to derive its z-score for the purposes of distance estimation. Second, we grouped the parameter sets into a binary, hierarchical cluster tree. To link pairs of parameter sets that are in close proximity we used the average linkage function. The average linkage function uses the unweighted average distance between all pairs of parameter sets to determine proximity between two parameter sets. As parameter sets are paired into binary clusters, the newly formed clusters are grouped into larger clusters until a hierarchical tree is formed. We verified that the linkage that we created is accurate, using the cophenetic correlation coefficient. Last, we cut the hierarchical tree such as the data are partitioned in the most dissimilar clusters, considering their cophenetic distance (measure of between-cluster dissimilarity) by visual inspection of the dendrograms.

We used AHC for the following reasons: (1) There is no assumption of a particular number of clusters, (2) any desired number of clusters can be obtained by 'cutting' the dendrogram at different levels, (3) AHC is used to find natural divisions in the data, (4) this algorithm uses a distance metric to create the cluster, making it ideal to separate the most dissimilar clusters, (5) it is the most commonly used unsupervised clustering algorithm and the method itself as well as its result are easily interpretable. Furthermore, AHC has been used to explore the parameter space and cluster model parameters in biological dynamical systems model reduction problems.[11] AHC has a few hindrances: First, it is to slow for large datasets as its runtime scales with an order of O(n2 log(n)), where n is the number of "individuals" to be clustered. This was not an issue our study as n was relatively low. Second, once a decision is made to combine two clusters, it can't be undone. This does not affect the purposes of our analysis that is not to identify the best hierarchical structure but rather the most dissimilar clusters. Last, AHC is characterized by sensitivity to noise and outliers. Although this does not interfere with the reason why we performed a clustering algorithm to the data (to identify dissimilar clusters), we did perform a sensitivity analysis and examined the hierarchical structure of the parameter space after excluding outliers, showing no significant change.

7. *Evaluation of our model development methodology by assessing its ability to recover the dynamic behaviors of the baseline action potential model.*

To evaluate the performance of our model development methodology, we applied it on the APDR intercept and slope of the baseline model, in a model recovery approach. We applied the same GA that we used with clinical data to the APDR derived from cell-level simulations of the baseline model and derived a population of

“recovered” baseline models (ReBASE). We show the parameter space bounds that we used in the final GA run (derived similarly to the bounds used in SNLV and ICMP model development) in Supplemental Table 6. We then performed AHC in the population of derived models to identify the most dissimilar clusters, similar to the methods that we describe in the clinical data. Last, we performed cell-level and tissue-level simulations using the centroids of the clusters identified by AHC in the ReBASE model population. We evaluated whether the emergent behaviors of the ReBASE models are similar to the baseline model.

Supplemental Results

Supplemental baseline characteristics. 10 patients (45.5%) had APDR data collected using a standard 4-mm tip ablation catheter and 12 (54.5%) using a multi-electrode catheter (10 (45.5%) with a Pentaray 4-4-4 catheter and 2 (9%) with a LiveWire catheter). The percentage of ICMP and SNLV patients that had APDR data collected using a multi-electrode ablation catheter vs. an ablation catheter was similar (for multi-electrode catheter 60% vs 50%, p=0.69). A total of 231 APDR curves were used in this analysis. 38 (16.5%) of those were collected using a standard 4-mm tip ablation catheter and 193 (83.5%) using a multi-electrode catheter (164 (71%) with a Pentaray 4-4-4 catheter and 29 (12.5%) with a LiveWire catheter). The percentage of APDR curves that were collected from ICMP and SNLV patients using a multi-electrode ablation catheter vs. an ablation catheter was similar (for multi-electrode catheter: 84.5% vs 82.8%, p=0.74).

Our model development methodology is robust in capturing the emergent dynamic behaviors in model-recovery analysis. The population of ReBASE models were successfully clustered using AHC. The cophenetic correlation coefficient was 0.98 for which suggests excellent hierarchical clustering. Clustering of the GA-derived ReBASE models was asymmetric, with the vast majority of models clustering in one large cluster (number of GA-derived models corresponding to the highest 4 branches of the dendrogram was 1507, 2, 2, and 1, Supplemental Figure 10D). In sensitivity analysis, when applying AHC to GA-derived ReBASE models with parameter values within the 99th and 95th percentile of the GA-derived model population the number of models corresponding to the highest 4 branches was: 1441, 9, 3 and 2 and 1056,109, 29, and 10 respectively. The action potential waveform of the ReBASE models was nearly identical to the waveform of the base model (Figure 10C). ReBASE models developed electrical alternans at 315-325 ms, which is consistent with what is observed in the to the baseline model (alternans at 325 msec; Supplemental Figures 10A and 10E). ReBASE models developed sustained functional re-entry over a very narrow range of S1S2 coupling intervals and only at the lowest conductivity values, which is consistent with what is observed in the baseline model, (Supplemental Figures 10B and 10F). These results support that our model development approach is valid, as it adequately captures the dynamic behavior of the baseline model in a model recovery approach.

Additional Considerations

<u>Spatial heterogeneity of APDR:</u> Both the left and the right ventricle exhibit significant APDR slope spatial heterogenety.[12] In this study, despite the fact that we

demonstrated an increased within-ventricle APDR slope variability in patients with ICMP compared to SNLV, we did not characterize the spatial heterogeneity of APDR. This was outside the scope of this study that had as a primary goal to clinically characterize the APDR of the surviving LV myocardium of ICMP patients and compare it to the APDR of SNLV. The exact locations within the LV where APDR was evaluated were not recorded. One potential limitation of this is the theoretical possibility that the inherent spatial APDR slope variability affects our results. However, we believe that such a possibility is merely theoretical rather than practical. The reasons for this are: First, the recording catheters were randomly placed in an endocardial location where viable myocardium was detected and thus the chances that we have selectively sampled areas of steep APDR in ICMP patients and less steep APDR in SNLV are slim. Second, we used multi-electrode catheters in an equal percentage of patients with ICMP and SNLV that enabled interrogation of sites in the left ventricle being on average 8-12mm away (based on the 4-4-4 inter-electrode spacing). Third, we analyzed the experimental findings using nested statistical analysis. Last the difference of APDR slope between ICMP and SNLV is significantly higher from what has been described between apex/base and LV/RV APDR slope difference in previous human studies.[12]

Applicability of our methodology to future studies: The signal processing and model development methods that we present in this study are directly generalizable to any action potential model and disease state that affects the APDR of the ventricles. We developed and used a semi-automatic, reproducible approach to assess ARI from clinical UniEGM recordings by combining and adapting different signal processing methods.[1-3] Reproducibility and automation are extremely important when an exact measurement is desired in noisy clinical recordings. We performed an extensive sensitivity analysis and optimization of our GA settings before applying our GA to develop action potential models from clinical data. Sensitivity analysis is critical when optimizing cardiac action potential models using only a subset of their properties (in this case APDR).[9] In the absence of sensitivity analysis the resulting GA solutions may exhibit considerable variability with respect to their emergent dynamics in simulations.[6] Finally, we combined machine learning with computer-based simulations capture the variability in the population of models derived from the GA. This is a novel application of machine learning with important implications for virtual heart modeling.

Use of GA in sampling the feasible parameter space for a given target: GAs have been used for sampling the feasible space of a model,[13] and their use in this context can be justified by the following reasons: First, the initial population of parameters is randomly drawn from a uniform distribution densely covering the parameter space. There is no bias or a-prior information that influences the initial population selection. Second, specifically in our study, we executed the GA 10x following the “local iterative” method, starting from different initial populations, and introducing different random mutations. By executing several GA iterations, each iteration samples and evolves over a different portion of the parameter space. Third,

GA extend methods of static parameter-space sampling such as Latin Hypercube sampling, as they evolve the initial uniformly sampled parameter space towards the target. The introduction of mutations prevents the GA evolution process to get trapped in local minima, which could limit the exploration of the parameter space.

Supplemental References

1. van Duijvenboden S, Orini M, Taggart P and Hanson B. Accuracy of measurements derived from intracardiac unipolar electrograms: A simulation study. *Conf Proc IEEE Eng Med Biol Soc*. 2015;2015:76-9.
2. Berger RD. QT variability. *J Electrocardiol*. 2003;36 Suppl:83-7.
3. Berger RD, Kasper EK, Baughman KL, Marban E, Calkins H and Tomaselli GF. Beat-to-Beat QT Interval Variability. *Circulation*. 1997;96:1557-1565.
4. Holland PW and Welsch RE. Robust regression using iteratively reweighted least-squares. *Communications in Statistics - Theory and Methods*. 1977;6:813-827.
5. Street JO, Carroll RJ and Ruppert D. A Note on Computing Robust Regression Estimates Via Iteratively Reweighted Least Squares. *The American Statistician*. 1988;42:152-154.
6. Cairns DI, Fenton FH and Cherry EM. Efficient parameterization of cardiac action potential models using a genetic algorithm. *Chaos*. 2017;27:093922.
7. Bueno-Orovio A, Cherry EM and Fenton FH. Minimal model for human ventricular action potentials in tissue. *J Theor Biol*. 2008;253:544-60.
8. Groenendaal W, Ortega FA, Kherlopian AR, Zygmunt AC, Krogh-Madsen T and Christini DJ. Cell-specific cardiac electrophysiology models. *PLoS Comput Biol*. 2015;11:e1004242.
9. Krogh-Madsen T, Jacobson AF, Ortega FA and Christini DJ. Global Optimization of Ventricular Myocyte Model to Multi-Variable Objective Improves Predictions of Drug-Induced Torsades de Pointes. *Front Physiol*. 2017;8:1059.
10. Vigmond EJ, Weber dos Santos R, Prassl AJ, Deo M and Plank G. Solvers for the cardiac bidomain equations. *Prog Biophys Mol Biol*. 2008;96:3-18.
11. Nienaltowski K, Wlodarczyk M, Lipniacki T and Komorowski M. Clustering reveals limits of parameter identifiability in multi-parameter models of biochemical dynamics. *BMC Syst Biol*. 2015;9:65.
12. Nash MP, Bradley CP, Sutton PM, Clayton RH, Kallis P, Hayward MP, Paterson DJ and Taggart P. Whole heart action potential duration restitution properties in cardiac patients: a combined clinical and modelling study. *Exp Physiol*. 2006;91:339-54.
13. Sen MK and Stoffa PL. Rapid sampling of model space using genetic algorithms: examples from seismic waveform inversion. *Geophysical Journal International*. 1992;108:281-292.

Supplemental Figures

Supplemental Figure 1

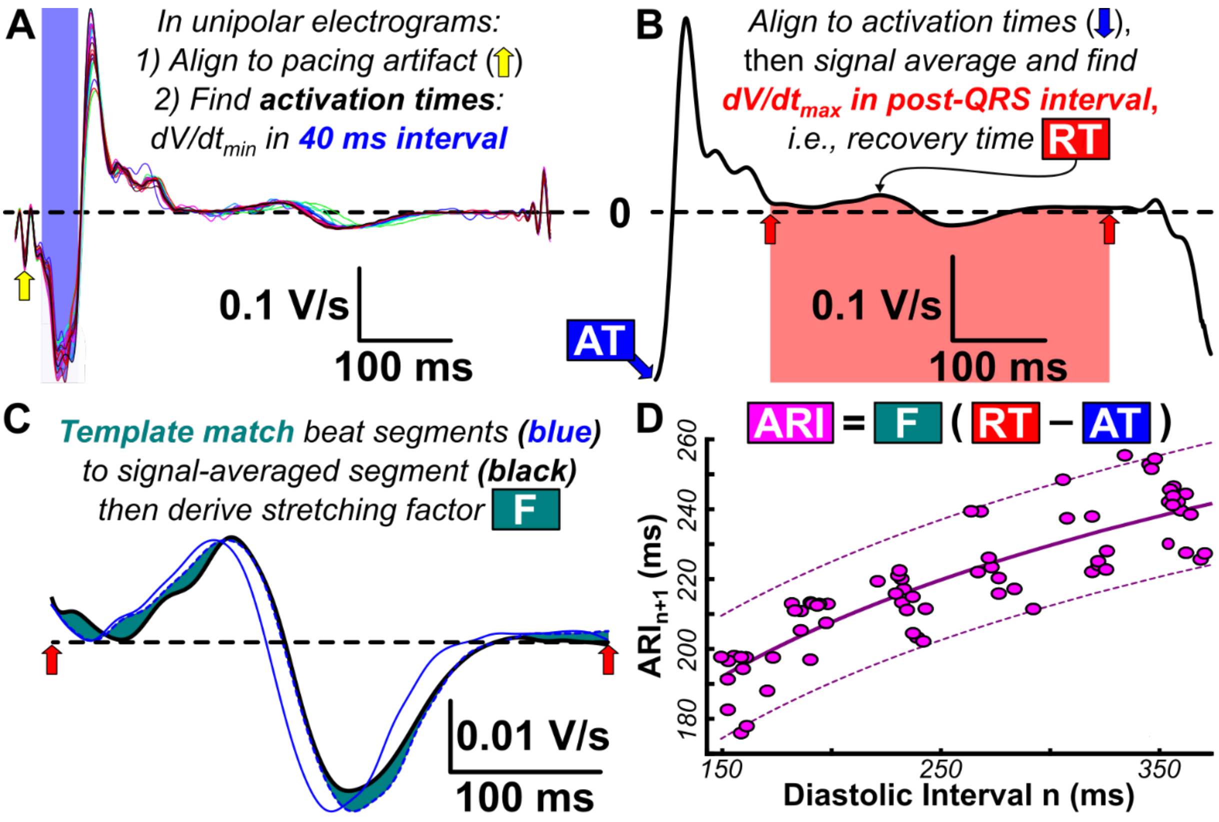

Supplemental Figure 1. Signal processing approach for reproducible and semi-automatic extraction of ARI from UniEGM time series. **A.** Waveforms represent the signals of different beats recorded from a single electrode of the recording catheter. The first derivative of the UniEGM time series is obtained (dV/dt) and all beats are aligned to the pacing artifact (yellow arrow). Activation time (AT) is defined in a search window of 40ms after the onset of the ventricular depolarization waveform on the UniEGM as the minimal dV/dt. **B.** the beats are subsequently aligned to the AT (blue arrow) and signal averaged. Recovery time (RT) in the signal-averaged beat is defined as the maximum dV/dt in a search window that extends from the offset of the ventricular depolarization UniEGM waveform to the next beat (red arrows). ARI of the signal averaged beat is the difference between RT and AT **C.** For each individual beat a stretch factor F is calculated such that when the individual beat is stretched in time by F the sum-squared error between the waveform of the *dV/dt* of that beat and the *dV/dt* of the signal-averaged beat (shaded area) is minimized. The ARI of each beat is calculated by multiplying the stretching factor of each beat with the ARI derived from the signal-averaged beat. **D.** APDR curve derived from logarithmic regression of the ARI of each beat on the DI of the previous beat. The solid line shows best-fit regression line using a bi-square regression approach, and interrupted lines show 95% confidence intervals.

Supplemental Figure 2

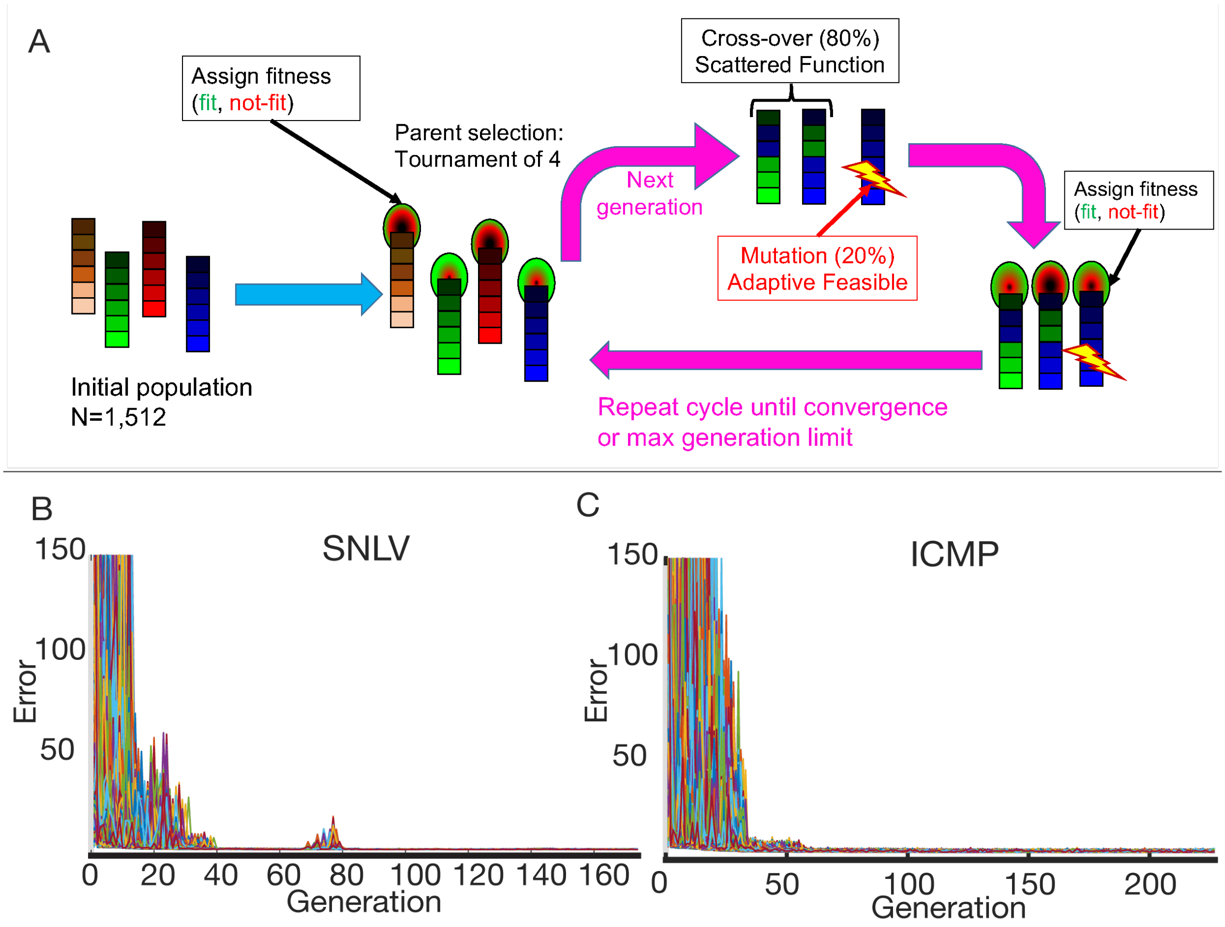

Supplemental Figure 2. A: Schematic of the genetic algorithm that we used for development of cohort-specific action potential models using clinically assessed APDR. We generated a random initial population of 1,512 parameter sets (individuals). Subsequently, we assessed these individuals for their fitness as described in the Supplemental Materials. The fittest individuals (green) were selected as parents and they gave rise to the next generation by cross-over (80%) or mutation (20%). Not-fit individuals gave were eliminated. This process was iteratively repeated until the algorithm converged, or the number of maximal allowed generations was reached. **B-C:** Convergence of the GA when used for development of SNLV (upper graph) and ICMP (lower graph) models. Each line in this graph represents an individual set of parameters as it evolved by the GA. The peaks observed at generation 70-80 during SNLV development are likely a result of the mutations introduced in the algorithm, driving the parameter sets to less fit solutions that were eventually eliminated as the GA continued to evolve,

Supplemental Figure 3

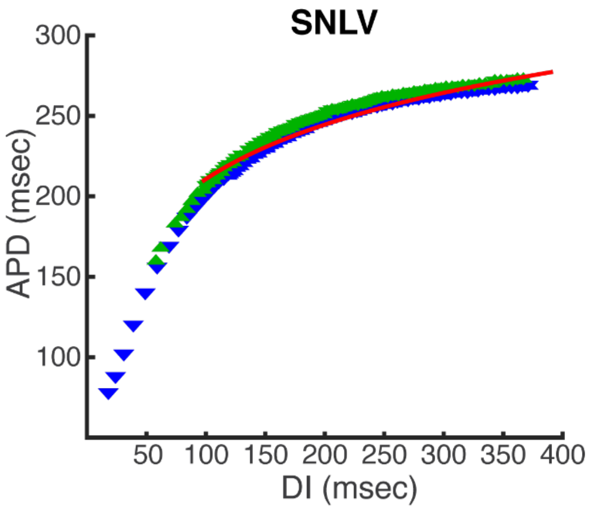

Supplemental Figure 3. APDR curve of SNLV and ICMP models derived from cell-level simulations (blue) and tissue-level simulations (green). The red line represents the fitted APDR curve to patient data for each cohort. The APDR curve derived from tissue-level simulations essentially overlaps with that derived from cell-level simulations. The restitution slope is identical between tissue-level and cell-level ADPR for both SNLV and ICMP. Tissue-level effective refractory period is reached at higher cycle length compared to cell-level effective refractory period.

Supplemental Figure 4

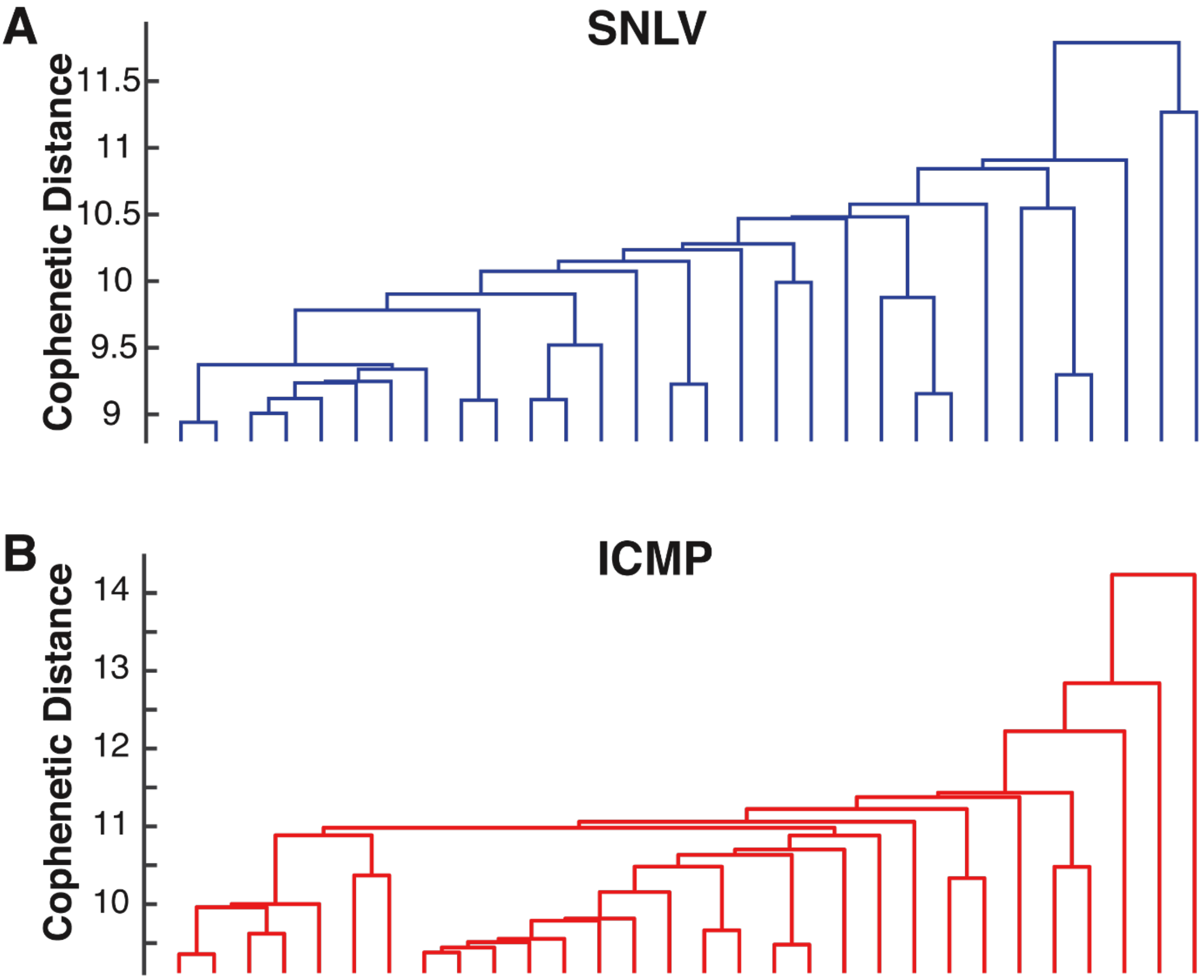

Supplemental Figure 4. Hierarchical clustering of the population of the GA-derived models produced by incorporating clinically assessed APDR in patients with **(A)** SNLV and **(B)** ICMP. The y-axis of the dendrograms is the cophenetic distance, which is a measure of dissimilarity between clusters. Each branch of the dendrogram represents an individual cluster. Only 30 levels of the histograms are presented. We selected to divide the population of GA-derived models based on the top 2 and 4 branches of the dendrogram. These branches had a significantly larger cophenetic distance compared to the lower-level branches, suggesting more dissimilarity between the individual clusters.

Supplemental Figure 5

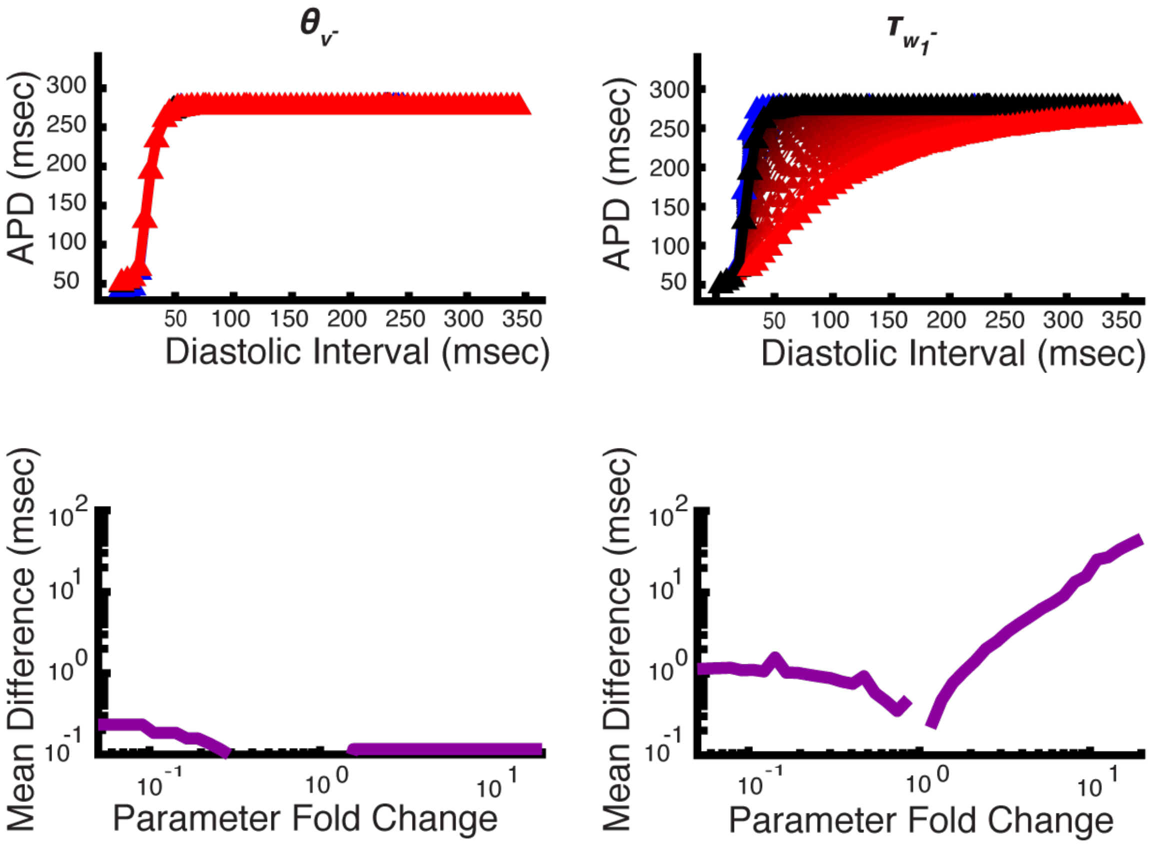

Supplemental Figure 5. Effect of variation of the parameters θ_v^- (left plots) and τ_w^- (right plots) on APDR as an example of our sensitivity analysis. Upper row shows APDR curve from cell-level simulations while increasing/decreasing the parameter of interest by 20 times on a logarithmic scale. Black line is the APDR curve for the baseline parameter value. Red lines represent APDR curves when the parameter is increased up to 20 times, with the lighter colors corresponding to greater increases. Blue lines represent APDR curves when the parameter is decreased by up to 20 times, with the lighter colors corresponding to greater decreases. The parameter θ_v^- yields the same APDR for the entire parameter spectrum. The lower rows show the mean difference of the APDR across changes in the parameter values. For θ_v^-, there is minimal change in APDR with variation of θ_v^- values, while for τ_w^- there is a considerable change in APDR with changes of τ_w^- value. As a result of this analysis, we excluded θ_v^- from the GA and maintained τ_w^-.

Supplemental Figure 6

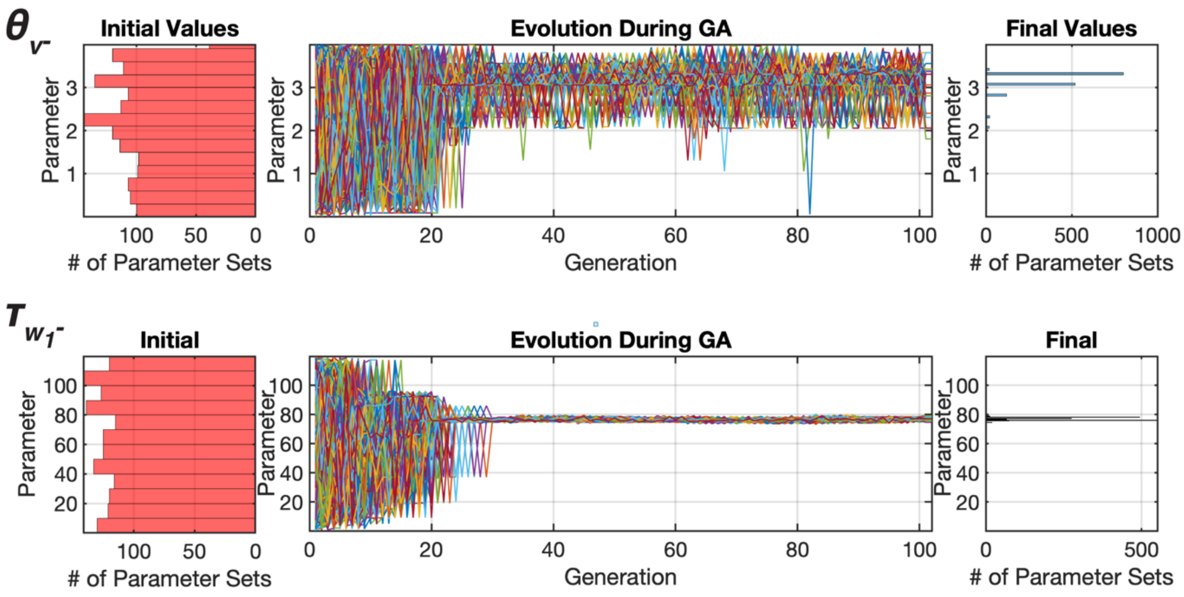

Supplemental Figure 6. Convergence test of the parameters θ_v^- (left plots) and τ_w^- (right plots) as an example of our sensitivity analysis. The upper panel shows the convergence of the GA for the parameter θ_v^- and lower row shows the convergence for the parameter τ_w^-. The left panel shows the uniform distribution of parameter values at the initial population of the GA. The middle panel shows value of the parameter during the evolution of the population by the GA (each line corresponds to a different individual). The right panel shows the distribution of the parameter value at the last generation yielded by the GA. For θ_v^- the parameter values do not converge, and the distribution of the parameter value is spread at the last generation. For τ_w^-, the parameter converges approximately after 30 generations. The distribution of τ_w^- in the last generation is narrow. As a result of this analysis, we excluded θ_v^- from the GA and maintained τ_w^-.

Supplemental Figure 7

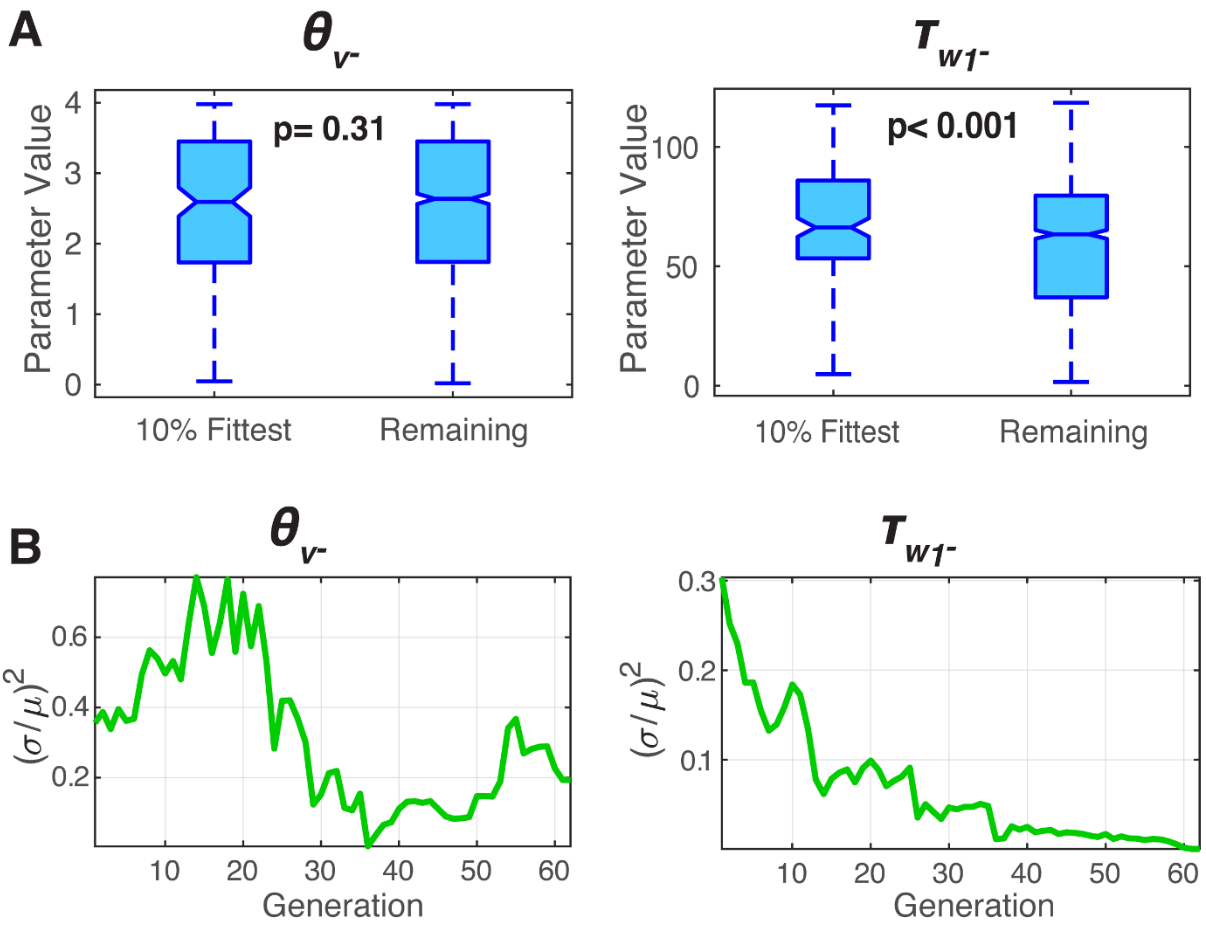

Supplemental Figure 7. A. Comparison between the 10% fittest individuals with the remaining individuals of the parameters θ_v^- (left) and τ_w^- (right) as an example of our sensitivity analysis. Box plots representing the distribution of the parameter value in the 10% fittest individuals of the 10th generation and the remaining individuals. The 10% fittest individuals have a significantly different distribution compared to the remaining individuals for τ_w^- (upper right plot), but not for θ_v^- (upper left plot). **B.** Variance convergence analysis of the parameters θ_v^- (left) and τ_w^- (right) as an example of our sensitivity analysis. X-axis represents generation during the GA evolution and Y-axis represents normalized variance of the parameter among all individuals in the same generation. There is convergence of normalized variance with progression of generations for τ_w^- (lower left plot) but not for θ_v^- (lower right plot). As a result of this analysis, we excluded θ_v^- from the GA and maintained τ_w^-.

Supplemental Figure 8

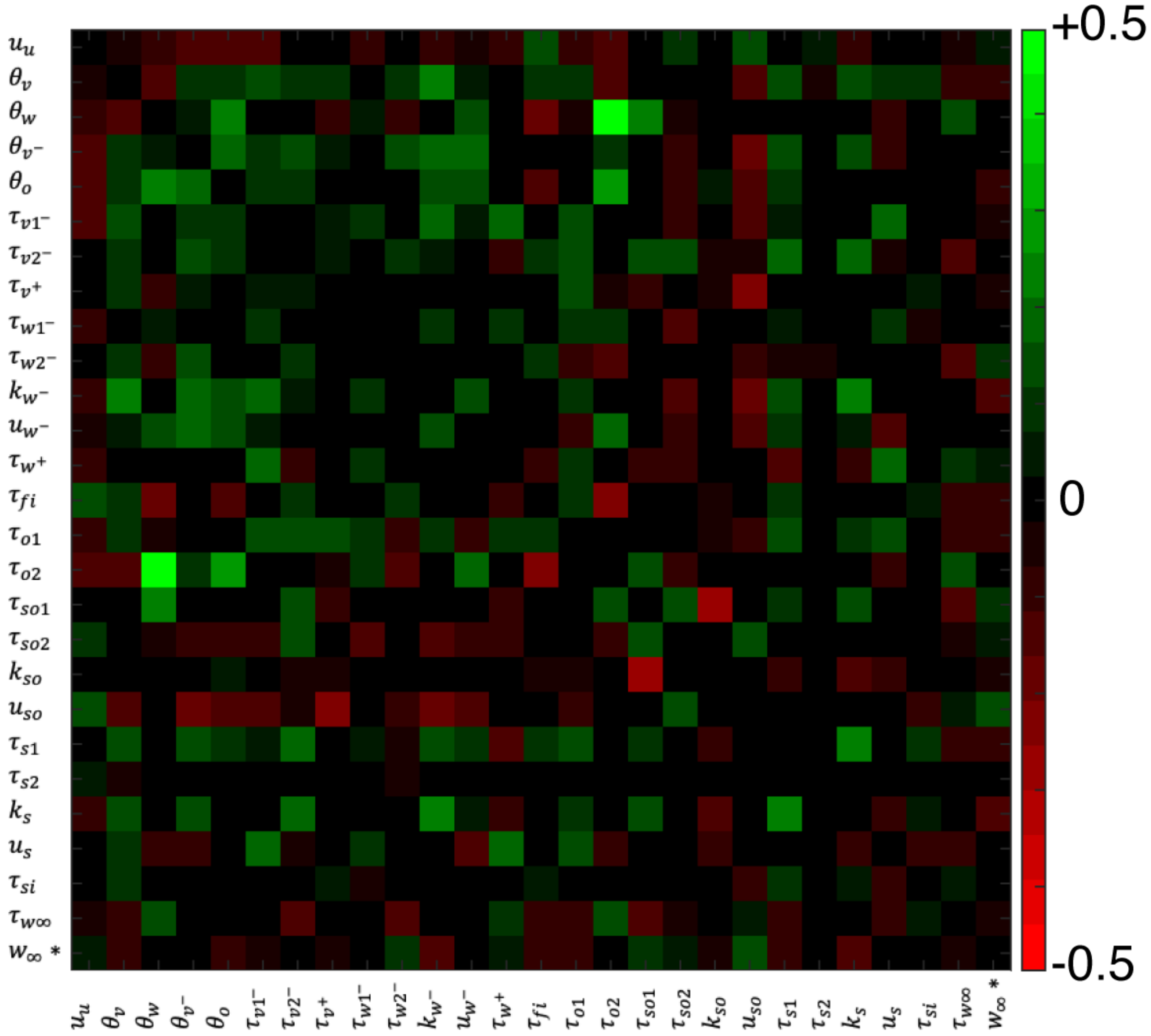

Supplemental Figure 8. Correlation matrix between all 27 parameters of the baseline model (resting membrane potential is included) of the 10th generation of a GA run during sensitivity analysis. Color scale represents correlation coefficient. From this analysis, the parameters τ_{o2} and θ_w should not co-exist in the same model development attempt. We excluded the parameter θ_w from our analysis, based on its performance on the other tests.

Supplemental Figure 9

A

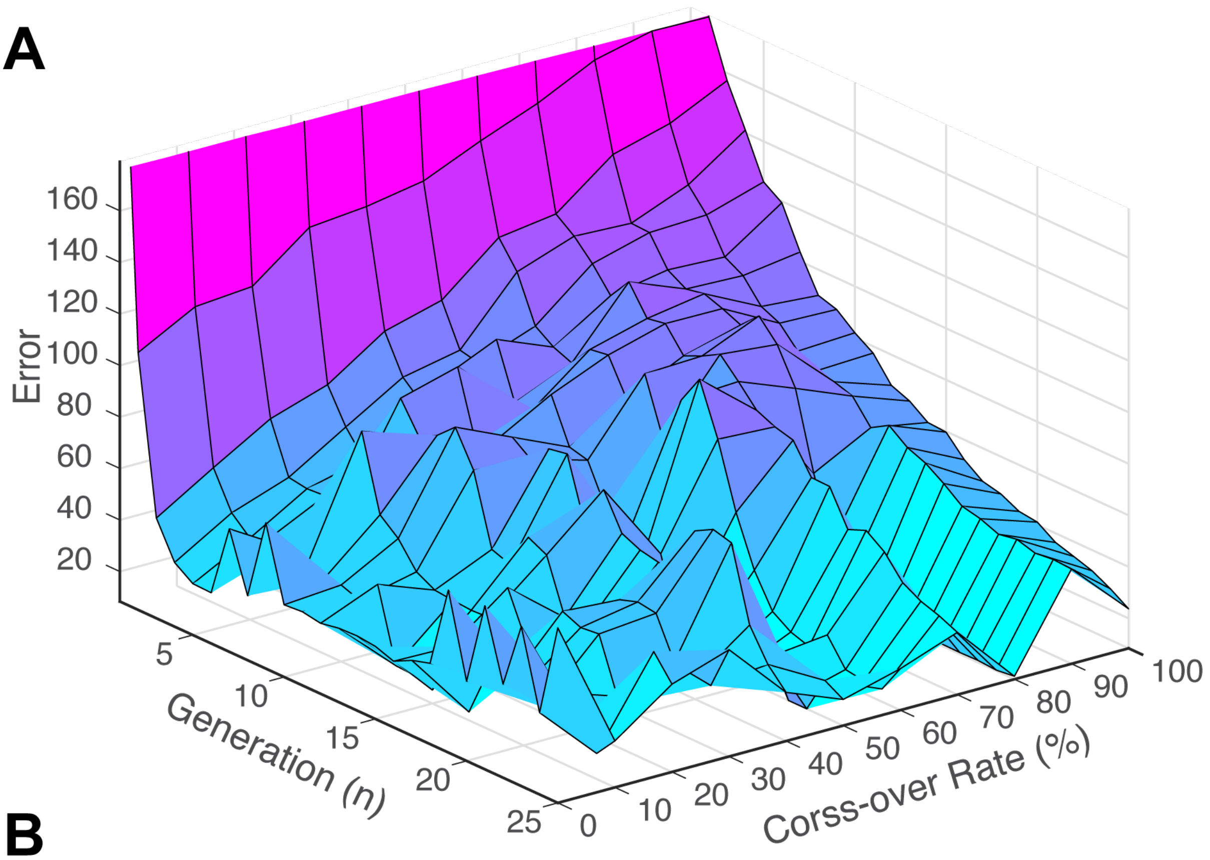

B

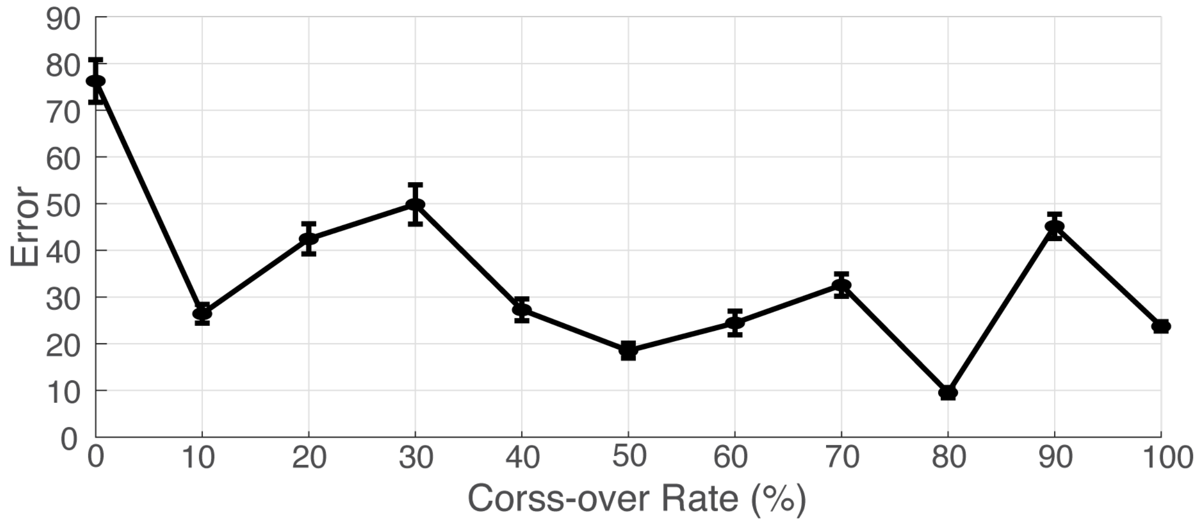

Supplemental Figure 9. Analysis to choose the optimal cross-over rate for the GA. A. We present the results of the 25 first generations of a GA run during development of SNLV models for cross-over rates that range from 0 to 100%. **A** cross-over rate of 80% results in steep and monotonic convergence. **B.** Mean error amongst all individuals of the 25th generation for cross-over rates that range from 0 to 100%. Error bars represent 95% confidence intervals of the mean error. A cross-over rate of 80% is associated with the lowest error. Based on this analysis we selected a cross-over rate of 80% for our GA.

Supplemental Figure 10

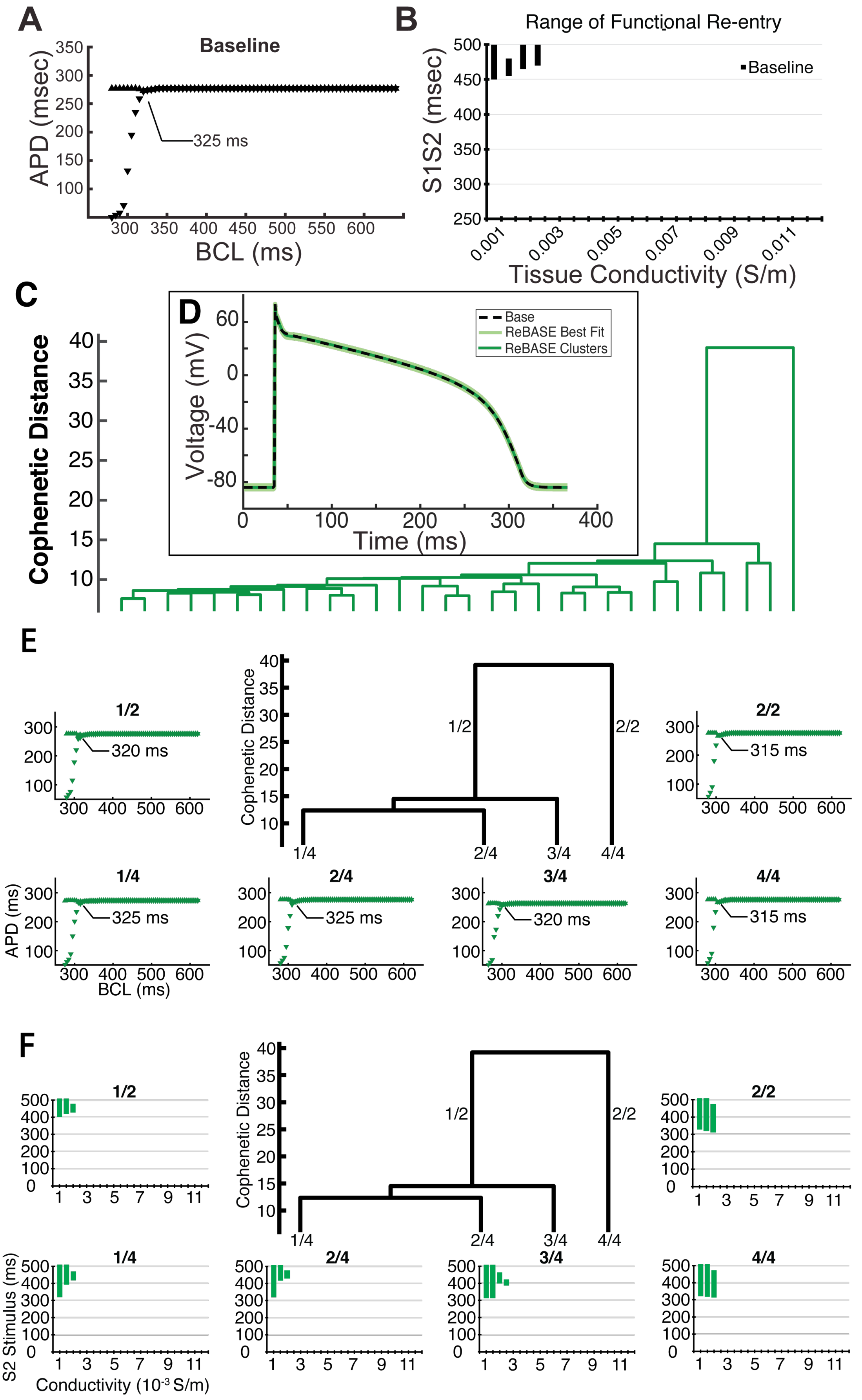

Supplemental Figure 10. A. Bifurcation plot of the baseline model derived from single cell simulations. The onset of repolarization alternans (development of bifurcation) is annotated. **B.** Range of functional re-entry inducibility in the baseline model. X-axis represents different tissue conductivity values in Siemens/m and Y-axis represents S1S2 coupling intervals that resulted in sustained functional re-entry. **C.** Action potential waveform of the base BOFC model (black line), best fit ReBASE model (dark green), and models using the centroids of the 6 highest clusters. Jitter has been applied to the waveforms using the centroids of the 6 highest clusters to facilitate visualization. **D.** Hierarchical clustering of the population of the GA-derived models during model recovery of the baseline model (ReBASE). Only 30 levels of the histograms are presented. **E.** Single-cell simulations in the 2 and 4 highest level clusters of ReBASE models. In the center of each sub-plot we show the 4 highest-level clusters of the dendrogram produced by AHC. The 2 highest-level clusters are noted as 2/1 and 2/2 and the 4 highest level clusters are noted as 4/1, 4/2, 4/3 and 4/4. Note that clusters 2/2 and 4/4 are identical since no bifurcation of the dendrogram occurs at this level. The bifurcation plots surrounding the dendrograms are labeled after the cluster that they have been created from (X-axis is pacing cycle length in ms and Y-axis is APD in ms). Cell level behavior of the most dissimilar clusters of the ReBASE exhibits the same dynamics with the baseline model. **F.** Tissue-level simulations in the 2 and 4 highest-level clusters of ReBASE models. In the center of each sub-plot we show the 4 highest-level clusters of the dendrogram produced by AHC. The 2 highest-level clusters are noted as 2/1 and 2/2 and the 4 highest clusters are noted as 4/1, 4/2, 4/3 and 4/4. Note that for clusters 2/2 and 4/4 are identical since no bifurcation of the dendrogram occurs at this level. Each plot surrounding the dendrogram shows the S1S2 coupling intervals that resulted in sustained functional re-entry for different conductivity values and is labeled after the cluster that it has been created from (X-axis represents different tissue conductivity values in mS/m and Y-axis represents S1S2 coupling intervals that resulted in sustained functional re-entry). Tissue level behavior of the most dissimilar clusters of the ReBASE exhibits the same dynamics with the baseline model

Supplemental Tables

Supplemental Table 1

Sensitivity analysis for differences in APDR intercept and slope between patients with SNLV and ICMP.

Sensitivity Analysis (Exclude)	APDR Slope Difference				APDR Intercept Difference			
	Mean		SEM	p-value	Mean		SEM	p-value
AF	34.9	±	10.1	0.001	-200.6	±	57.5	<0.001
AAD Class III	31.9	±	10.4	0.002	-184.9	±	64.5	0.004
AAD Class III + Ranolazine	30.9	±	12.4	0.013	-177.1	±	77.6	0.022
AAD Class III + Class I + Ranolazine	32.9	±	12.5	<0.001	-182.7	±	78.3	0.020
Patients with highest and lowest slopes	17.9	±	6.28	0.005	-105.7	±	34.7	0.002
Women	25.4	±	11.5	0.027	-163.2	±	65.8	0.013
R^2<60%	30.8	±	9.9	0.002	-181.7	±	56.8	0.001
R^2<70%	30.5	±	9.2	0.001	-180.6	±	52.6	0.001
R^2<80%	31.1	±	10.0	0.002	-188.9	±	57.1	0.001
R^2<90%	37.7	±	12.7	0.003	-222.5	±	78.2	0.004

Results are reported as mean ± standard error of the mean. P-values are from mixed model analysis after excluding patients with the properties listed on the first column of the table. Abbreviations: AAD: anti-arrhythmic drugs, AF: atrial fibrillation, R^2: coefficient of determination of the logarithmic curve fit to individual APDR curves.

Supplemental Table 2

Parameter set for the GA-derived SNLV model.

Parameter	SNLV min bound	SNLV max bound	SNLV Final
τ_{v^+}	0.336	2.878	1.882
τ_{w1^-}	94.224	99.944	99.864
τ_{w2^-}	124.820	148.013	147.080
k_{w^-}	11.985	56.066	17.793
τ_{w^+}	154.278	263.140	163.538
τ_{o2}	2.358	9.574	7.591
τ_{so1}	25.821	66.134	50.632
τ_{so2}	0.515	4.952	2.354
k_{so}	1.348	3.238	2.340
u_{so}	0.870	1.000	0.987
τ_{s2}	4.419	12.509	7.661
k_s	3.320	9.710	7.675
u_s	0.584	0.836	0.738
τ_{si}	3.882	18.995	9.210
w_∞ *	0.726	1.000	0.986

This table presents the minimum and maximum bounds that we used in the final GA run to develop SNLV models. We derived SNLV min and max bounds by running the GA 10x as described in the supplemental material and methods. *SNLV Final* is the best-fit parameter of the SNLV model derived by the GA.

Supplemental Table 3

Parameter set for best-fit ICMP model.

Parameter	ICMP min bound	ICMP max bound	ICMP Final
τ_{v^+}	0.975	2.973	1.079
τ_{w1^-}	99.137	99.998	99.962
τ_{w2^-}	146.464	149.916	149.502
k_{w^-}	10.255	24.555	11.061
τ_{w^+}	260.501	298.309	295.619
τ_{o2}	9.531	9.940	9.830
τ_{so1}	15.956	68.894	63.892
τ_{so2}	1.888	4.289	2.038
k_{so}	1.182	2.122	1.303
u_{so}	0.878	0.992	0.932
τ_{s2}	4.075	13.654	5.374
k_s	2.990	9.998	8.338
u_s	0.632	0.884	0.768
τ_{si}	4.322	18.544	17.581
w_∞ *	0.400	0.442	0.401

This table presents the minimum and maximum bounds that we used in the final GA run to develop ICMP models. We derived ICMP min and max bounds by running the GA 10x as described in the supplemental material and methods. *ICMP Final* is the best-fit parameter of the ICMP model derived by the GA.

Supplemental Table 4

Parameters of the baseline model.

Parameter	ENDO
u_o	0
u_u	1.56
θ_v	0.3
θ_w	0.13
θ_{v^-}	0.2
θ_o	0.006
τ_{v1^-}	75
τ_{v2^-}	10
τ_{v^+}	1.4506
τ_{w1^-}	6
τ_{w2^-}	140
k_{w^-}	200
u_{w^-}	0.016
τ_{w^+}	280
τ_{fi}	0.1
τ_{o1}	470
τ_{o2}	6
τ_{so1}	40
τ_{so2}	1.2
k_{so}	2
u_{so}	0.65
τ_{s1}	2.7342
τ_{s2}	2
k_s	2.0994
u_s	0.9087
τ_{si}	2.9013
$\tau_{w\infty}$	0.0273
w_∞ *	0.78

Supplemental Table 5

Parameters of the baseline model that were varied in the GA to develop SNLV and ICMP models.

Parameter	Min bound	Max bound
τ_{v^+}	0.1	3
τ_{w1^-}	5	100
τ_{w2^-}	5	150
k_{w^-}	10	200
τ_{w^+}	100	300
τ_{o2}	2	10
τ_{so1}	10	100
τ_{so2}	0.1	5
k_{so}	1	10
u_{so}	0.3	1
τ_{s2}	2	20
k_s	1	10
u_s	0.1	1
τ_{si}	1	20
w_∞ *	0.4	1

15/28 parameters were selected for optimization after the sensitivity analysis as we describe in the supplemental material and methods. This table demonstrates the minimum and maximum bounds that were used in the 10x initial GA runs for each one of the baseline parameters that we optimized.

Supplemental Table 6

Parameter set used in the model-recovery analysis.

Parameter	ReBASE min bound	ReBASE max bound	ReBASE Final
τ_{v^+}	1.344	2.922	2.503
τ_{w1^-}	5.038	13.822	8.834
τ_{w2^-}	7.421	106.949	84.263
k_{w^-}	73.566	194.415	168.132
τ_{w^+}	106.511	180.784	110.815
τ_{o2}	4.431	6.463	5.768
τ_{so1}	17.429	71.401	48.223
τ_{so2}	0.797	4.365	0.857
k_{so}	2.028	3.178	3.088
u_{so}	0.843	1.000	0.971
τ_{s2}	2.977	8.961	6.502
k_s	2.508	9.935	4.568
u_s	0.301	0.801	0.686
τ_{si}	1.947	10.993	4.598
w_∞ *	0.479	0.998	0.556

This table presents the minimum and maximum bound that were used in the final GA run to fit the baseline model to APDRs derived from simulations of the baseline model (model recovery analysis). We derived ReBASE min and max bounds by running the GA 10x as described in the supplemental material and methods. ReBASE Final best-fit parameter of the ReBASE model derived by the GA.
